# PVDF and P(VDF-TrFE) Electrospun Scaffolds for Nerve Graft Engineering: A Comparative Study on Piezoelectric and Structural Properties, and In Vitro Biocompatibility

**DOI:** 10.3390/ijms222111373

**Published:** 2021-10-21

**Authors:** Oleksandr Gryshkov, Fedaa AL Halabi, Antonia Isabel Kuhn, Sara Leal-Marin, Lena Julie Freund, Maria Förthmann, Nils Meier, Sven-Alexander Barker, Kirsten Haastert-Talini, Birgit Glasmacher

**Affiliations:** 1 Institute for Multiphase Processes, Leibniz University Hannover, An der Universität 1, Building 8143, 30823 Garbsen, Germany; kuhn@imp.uni-hannover.de (A.I.K.); lealmarin@imp.uni-hannover.de (S.L.-M.); barker@imp.uni-hannover.de (S.-A.B.); glasmacher@imp.uni-hannover.de (B.G.); 2 Lower Saxony Centre for Biomedical Engineering, Implant Research and Development, Stadtfelddamm 34, 30625 Hannover, Germany; 3 Institute of Neuroanatomy and Cell Biology, Hannover Medical School, Centre for Systems Neuroscience (ZSN) Hannover, 30559 Hannover, Germany; freund.lena@gmail.com (L.J.F.); maria.foerthmann@gmx.de (M.F.); Haastert-Talini.Kirsten@mh-hannover.de (K.H.-T.); 4 Institute for Technical Chemistry, Braunschweig University of Technology, Hagenring 30, 38106 Braunschweig, Germany; nils.meier@tu-bs.de

**Keywords:** polyvinylidene fluoride, polyvinylidene fluoride-co-trifluoroethylene, electrospinning, scaffold, piezoelectric module, dynamic impact machine, peripheral nervous system, nerve conduit, in vitro performance, neurite outgrowth, zeta potential

## Abstract

Polyvinylidene fluoride (PVDF) and its copolymer with trifluoroethylene (P(VDF-TrFE)) are considered as promising biomaterials for supporting nerve regeneration because of their proven biocompatibility and piezoelectric properties that could stimulate cell ingrowth due to their electrical activity upon mechanical deformation. For the first time, this study reports on the comparative analysis of PVDF and P(VDF-TrFE) electrospun scaffolds in terms of structural and piezoelectric properties as well as their in vitro performance. A dynamic impact test machine was developed, validated, and utilised, to evaluate the generation of an electrical voltage upon the application of an impact load (varying load magnitude and frequency) onto the electrospun PVDF (15–20 wt%) and P(VDF-TrFE) (10–20 wt%) scaffolds. The cytotoxicity and in vitro performance of the scaffolds was evaluated with neonatal rat (nrSCs) and adult human Schwann cells (ahSCs). The neurite outgrowth behaviour from sensory rat dorsal root ganglion neurons cultured on the scaffolds was analysed qualitatively. The results showed (i) a significant increase of the β-phase content in the PVDF after electrospinning as well as a zeta potential similar to P(VDF-TrFE), (ii) a non-constant behaviour of the longitudinal piezoelectric strain constant *d*_33_, depending on the load and the load frequency, and (iii) biocompatibility with cultured Schwann cells and guiding properties for sensory neurite outgrowth. In summary, the electrospun PVDF-based scaffolds, representing piezoelectric activity, can be considered as promising materials for the development of artificial nerve conduits for the peripheral nerve injury repair.

## 1. Introduction

Peripheral nerve injuries (PNIs) occur in about 2.8% of trauma patients [1] reflecting its treatment to be of great importance as the patients suffer from life-long symptoms such as neuropathic pain and the loss or disturbances in sensory and motor function [2,3]. In case of a severe loss of nerve tissue (>0.5 cm), the use of autologous nerve tissue has emerged as the ‘gold standard’ procedure for bridging critical distances (0.5–6.0 cm) [4,5]. Despite being the standard procedure, there are several disadvantages associated with the use of autologous nerve grafts: a need for secondary surgery, damage at the donor site including sensory loss and scarring, restrictions to the amount of donor tissue, a limited functional recovery and implant-associated infections [6,7,8]. In this regard, the development and application of biosynthetic nerve guidance conduits (NGCs) represents a huge potential for repairing and regenerating PNIs [9].

The materials intended for the utilisation to engineer the NGCs have to combine several advantageous characteristics in order to compete with the gold standard. They should be biocompatible, biodegradable without the release of toxic degradation products, mechanically flexible, but collapse resistant [4]. In addition, the prospective materials should ensure a barrier function and semipermeability for the exchange of nutrients as well as to provide cues for an axonal cell ingrowth. Among the natural materials to engineer the NGCs, biopolymers such as chitosan, collagen, fibrin, and agarose have been widely used [10,11,12]. In turn, synthetic polymers, such as polylactic acid, poly(ethylene oxide), poly(ε-caprolactone) (PCL), and others have been utilised to develop NGCs and showed promising results for the PNIs repair (sciatic nerve defect) in vivo [13,14]. Along with contradicting information on the immune response of some synthetic polymers [13,14,15], these materials cannot provide sufficient, if any, electric activity for the stimulation of the Schwann cells (SCs) ingrowth. Thus, to start the regeneration processes, biochemical and electric signals from the injury site of axons have to be transported to their cell body to promote a change in gene regulation. The porous biocompatible materials possessing piezoelectric activity, such as polyvinylidene fluoride (PVDF) and its copolymer polyvinylidene fluoride-trifluoroethylene (P(VDF-TrFE)), could be advantageous to guide growing neurites and migrating SCs [16,17,18,19].

Due to their unique properties, electrospun porous scaffolds could mimic the extracellular matrix at the site of transplantation [20,21]. The main characteristics of the electrospun scaffolds, such as the fibre and pore size, thickness, and porosity, are precisely controllable by adjusting the process parameters including the voltage, spinning distance, polymer concentration, and electrospinning time. Moreover, this method allows to obtain uniaxially aligned fibres in a controlled manner by adjusting the rotation speed of a drum collector [22,23]. This offers additional benefits for the neuronal tissue engineering. In this regard, a recent review by Li et al. highlighted the prospective application of electrospun PVDF and P(VDF-TrFE) scaffolds for the bone and neural tissue engineering [24]. Several studies have confirmed the tissue regeneration supporting properties of the electrospun scaffolds made of PVDF-based polymers for bone [25], muscle [26], cardiovascular [27,28], lung [29] and, more notably, nervous [30,31] tissue engineering applications. The PVDF-based materials were shown to improve the axonal regeneration for central nerve injuries and PNIs [30], the neurite outgrowth of sensory DRG neurons [32,33], and the neural differentiation of stem cells [33].

The piezoelectric properties of PVDF-based materials predominantly result from the presence of the polar, crystalline β-phase, and can be characterised by the longitudinal piezoelectric strain constant (hereafter as piezoelectric module) *d*_33_ (the numbers indicate that the direction of the mechanical action and the electric signal measurement are along the same axis). Surmenev et al. have recently highlighted the most recent advances in the application of piezoelectrically active PVDF and P(VDF-TrFE) materials for biomedical sensing applications [34]. In contrast to the costly P(VDF-TrFE), where the β-phase content can be adjusted using different polymerisation ratios of VDF with TrFE, PVDF represents a cheaper alternative possessing, however, a high content of a non-polar α-phase. During the electrospinning, the α-phase in PVDF undergoes its transformation to β-phase due to the electrical poling. Thus, PVDF exhibits more flexibility in the production of piezoelectric PVDF-based scaffolds for tissue engineering applications with the desired β-phase content by varying the process parameters of the electrospinning, such as the electric field strength and a post-electrospinning temperature treatment [35,36,37], to name a few. In our previous study, we have reported on the measurement of the voltage, generated upon the mechanical loading of PVDF and P(VDF-TrFE) electrospun scaffolds using a static impact machine [38]. It is based on the manual Berlincourt method [39]. In this regard, Longbiao et al. have compared quasi-static, laser interferometry, and dynamic resonant systems for the measurement of the *d*_33_ constant of piezoelectric solid materials [40]. However, a sophisticated dynamic approach has still to be developed and validated to provide a dynamic loading and automated measurement of the generated voltage and to ensure a comparative analysis of the electrospun porous scaffolds with piezoelectric activity and their prospective application within the development of NGCs suitable for PNI repair.

Thus, the current work focuses on the analysis of PVDF and P(VDF-TrFE) electrospun scaffolds in terms of their processability by electrospinning, analysis of structural properties, and a direct piezoelectric effect using the developed dynamic impact loading machine. Utilising two SCs types (neonatal rat (nrSCs) as well as adult human SCs (ahSCs)) and cultivating sensory dorsal root ganglion (DRG) neurons on the scaffolds, we evaluated the biocompatibility, SC morphology on the scaffolds, and neurite outgrowth behaviour to suggest that these polymers represent promising candidates for the future engineering of artificial nerve conduits for PNI repair.

## 2. Results

### 2.1. Effect of the Polymer Type and Concentration on the Scaffold Morphology

**Figure 1:**
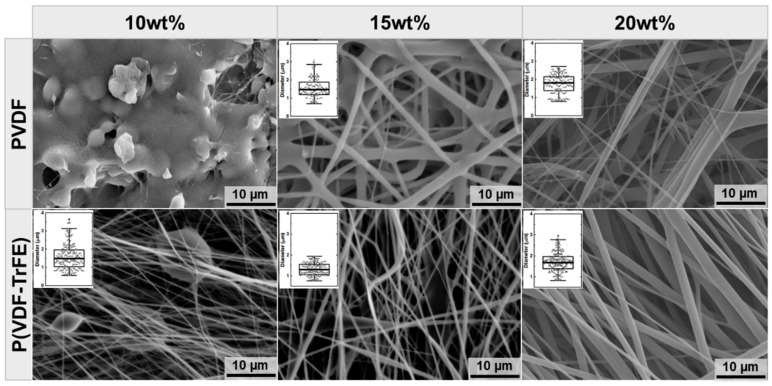
SEM images of PVDF and P(VDF-TrFE) electrospun scaffolds produced from the solutions of different polymer concentrations. The inserts represent the box plots (*Y*-axis scale is equal: from 0 to 4 µm with a step of 1 µm) of the fibre diameter distribution: open circles—single measurements; box—25–75% data range; line in the box—median; whiskers—1.5IQR. The scale bars are 10 µm.

The PVDF and P(VDF-TrFE) scaffolds were produced using the solution electrospinning in a horizontal orientation [41]. Figure 1 shows the scanning electron microscopy (SEM) images of the electrospun scaffolds made from 10, 15, and 20 wt% PVDF and P(VDF-TrFE) dissolved in *N*,*N*-dimethylformamide, and acetone (6:4). Our recent study demonstrated that the combination of these solvents in a volume ratio of 6:4 results in the production of PVDF-based scaffolds having the highest β-phase content and crystallinity [42]. The scaffolds produced from 10 wt% PVDF did not exhibit a fibrous morphology and thus were not considered for further investigations. The median fibre diameter of the electrospun P(VDF-TrFE) scaffolds was as follows: 1.49 (10 wt%), 1.29 (15 wt%), and 1.69 µm (20 wt%). In turn, the median of the fibre diameters of PVDF was calculated to be the following: 1.48 and 1.81 µm for 15 wt% and 20 wt% concentrations, respectively. Except for the 10 wt% P(VDF-TrFE) scaffolds containing beads of about 8 µm in diameter, the quantification of the SEM images revealed an increase in the fibre diameter of the electrospun scaffold with an increasing solution concentration.

**Figure 2:**
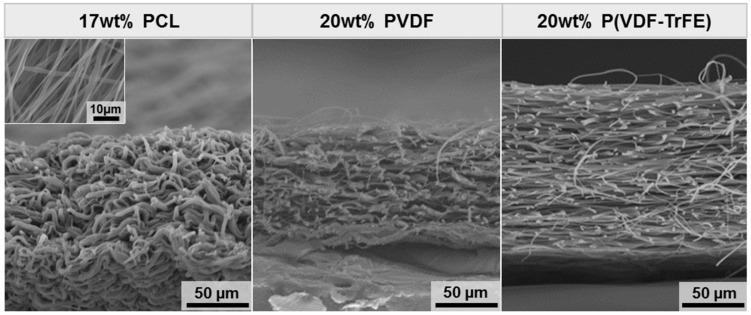
Representative SEM images of the cross-sectional view of the electrospun scaffolds. An insert to 17 wt% PCL shows the scaffold morphology. The scale bars are 10 (insert) and 50 µm.

By increasing the concentration of PVDF and P(VDF-TrFE), the electrospinning process becomes more stable. For this reason, the electrospun fibre mats with a concentration of 20 wt% were chosen for the zeta potential measurements, structural analysis, and cell studies. As a non-piezoelectric material, electrospun scaffolds produced from a 17 wt% PCL solution (median fibre diameter of 1.71 µm) were utilised as a control. Figure 2 shows the representative SEM images of the scaffolds’ cross-sections. The following values for the scaffolds’ thickness were obtained: 92 ± 6 (17 wt% PCL), 91 ± 10 (20 wt% PVDF), and 114 ± 18 µm (20 wt% P(VDF-TrFE)).

### 2.2. Effect of the Electrospinning on the Structural Changes and Phase Transformation

**Figure 3:**
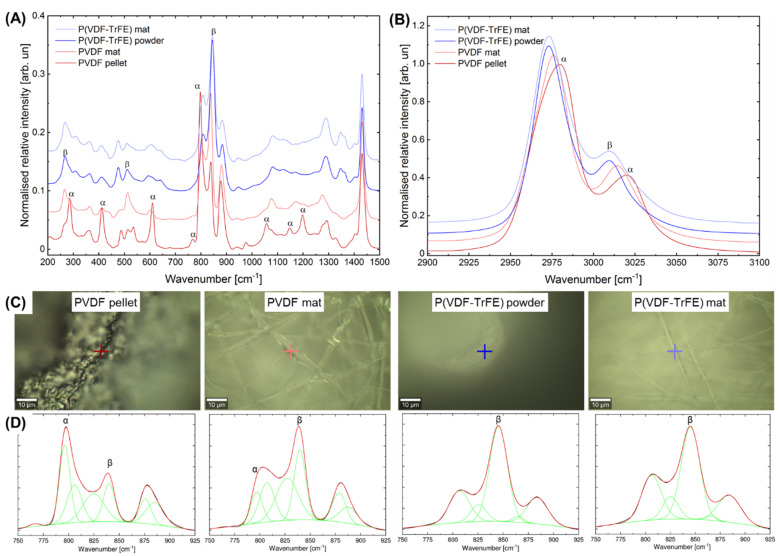
Raman microscopy of the initial materials and produced fibre mats. (**A**,**B**) Normalised Raman spectra of the initial materials (PVDF pellet and P(VDF-TrFE) powder) as well as the electrospun fibre mats in the fingerprint region ((**A**), 200–1500 cm^−1^) and in the region of the CH_2_ stretching ((**B**) 2900–3100 cm^−1^). (**C**) Photographs of the respective materials with the indicated points for the spectra acquisition. (**D**) Representative results of the Gaussian deconvolution of the Raman peaks in the region of 740–930 cm^−1^. The scale bars on (**C**) are 10 µm.

Raman spectroscopy was conducted to analyse the changes within the structure and phase composition of PVDF (α- and β-phase content) and P(VDF-TrFE) upon electrospinning. Figure 3 shows the representative Raman spectra of the initial materials (PVDF pellets and P(VDF-TrFE) powder) as well as the fibre mats in the wavenumber ranges of 200–1500 (Figure 3A) and 2900–3100 cm^−1^ (Figure 3B). Figure 3C highlights representative photographs of the sample surface and the respective position of the spectral acquisition. As can be seen, the main Raman bands of PVDF and P(VDF-TrFE) can be detected in two main regions, e.g., 2900–3100 and 200–1500 cm^−1^. Table 1 shows the main assignments of the bands presented within the Raman spectra of PVDF and P(VDF-TrFE). The first region is described by the symmetric (peak around 2975 cm^−1^) and antisymmetric stretching vibration (peak around 3011 cm^−1^) of CH_2_ groups. The fingerprint region is represented by two subregions in the ranges of 200–800 and 800–1500 cm^−1^, which contains peaks of different intensities corresponding to the molecular vibrations within the PVDF, non-polar α- and polar crystalline β-phases. The first subregion is mainly dominated by the presence of the Raman bands related to the molecular vibrations of the CF_2_ group (twisting, rocking, scissoring, and wagging), whereas a few peaks in this subregion can be attributed to the scissoring of the CCC group. The second subregion is dominated by the symmetric and antisymmetric stretching of the CF_2_ and CC groups, whereas some peaks corresponding to the CH_2_ rocking, wagging, and scissoring can also be detected. In the second main region of the Raman spectra of PVDF and P(VDF-TrFE) in the wavenumber range of 2900–3100 cm^−1^ (Figure 3B), two intense peaks at around 2980 cm^−1^ (including a shoulder at around 2971 cm^−1^) and 3021 cm^−1^ were observed, which can be assigned to the symmetric and antisymmetric stretching of CH_2_ groups, respectively.

Comparing the Raman spectra of P(VDF-TrFE) before and after the electrospinning, no noticeable changes in the main bands’ positions and intensities were observed. In contrast to P(VDF-TrFE), the Raman spectra of PVDF electrospun fibre mats changed noticeably. They exhibit an increase in the main bands’ intensity corresponding to the β-phase in PVDF (839 cm^−1^) and the respective decrease of the band at 797 cm^−1^ associated with the α-phase. After the electrospinning of PVDF, the prominent peaks of the α-phase (286, 412, 610, 797, 1057, 1148, 1198, and 1293 cm^−1^) decrease in intensity or even disappear. As can be seen, the Raman spectrum of the PVDF fibre mats is similar to that of the P(VDF-TrFE) materials. Figure 3B shows the region of the CH_2_ stretching, where two prominent peaks at 3021 and 2976 cm^−1^ are shifted towards lower wavenumbers of 3015 and 2970 cm^−1^, respectively, which underlines the transformation of the α- to β-phase.

Figure 3D shows the results of the Gaussian peak deconvolution in the wavenumber range of 740–930 cm^−1^ for the initial materials (P(VDF-TrFE) powder and PVDF pellet) as well as the respective electrospun fibre mats. In general, three typical peaks at around 790–810, 839–845, and 870–890 cm^−1^ were detected for the PVDF and P(VDF-TrFE) materials before and after electrospinning. This can be attributed to the CH_2_ rocking (α-phase), CH_2_ rocking, and CF_2_ antisymmetric stretching as well as the CC antisymmetric and CF_2_ symmetric stretching, respectively (Table 1). For the PVDF materials, an additional band at 797 cm^−1^ corresponding to the α-phase was detected. In order to analyse the transformation of the α- to β-phase within the PVDF due to the electrospinning, six spectra were acquired followed by a peak deconvolution and calculation of the β/α-phase ratio, based on the intensities of the respective bands at 839 (polar β-phase) and 797 cm^−1^ (non-polar α-phase). The results on the calculation of the β/α ratio (Table 2) show a noticeable increase in the β-phase content after the electrospinning (2.3 ± 0.6), as compared to the raw PVDF (0.4 ± 0.1). Regarding the electrospun PVDF mats, the intensity and area of the band at 797 cm^−1^ decrease yielding an increase in the intensity and area of the band at 839 cm^−1^, whereas the respective values of a full width at half maximum (FWHM) remain unaltered. This phenomenon results from the mechanical stretching and fast solvent evaporation during the electrospinning process, contributing to the alignment of dipole moments yielding a α- to β-phase transformation [37,43]. For the P(VDF-TrFE) materials, no noticeable changes in the intensity, area, and FWHM of the main band at 845 cm^−1^ attributed to β-phase were observed (Table 1). The representative results of the deconvolution of the Raman bands in the region of 740–930 cm^−1^ can be consulted in the Supplementary Materials (Figures S1–S4).

**Table 1:** Assignment of the main Raman bands of PVDF and its copolymer P(VDF-TrFE).

Raman Peak Position, cm^−1^				Peak Assignment	Ref.
PVDF Pellet	PVDF Fibre Mat	P(VDF-TrFE) Powder	P(VDF-TrFE) Fibre Mat		
266 (shoulder)	266	268	268	*α* CF_2_ t; CF_2_ w	[44]
286	286	285 (shoulder)	285 (shoulder)	*α* CF_2_ t; CF_2_ w	
363	365	365	365	n.a.	
412	412	411	411	*α* CF_2_ r; CF_2_ r	
488	480–488	476	476	CF_2_ δ; CF_2_ w	
513	513	511	511	*α* CF_2_ δ	
536	534 (shoulder)			*α* CF_2_ δ	
610	603	600–610	590–615	*α* CF_2_ δ; CCC δ	
		642	643	n.a.	
768				*α* CF_2_ δ; CCC δ	
797	790–812			*α* CH_2_ r	[45]
		807	807	*β* CH_2_ r	[44]
839	839			CH_2_ r; CF_2_ ν_a_	
		845	845	β CF_2_ ν_s_	[45]
878	881	883	884	*α* CC ν_a_; CF_2_ ν_s_ *β CC* ν_a_*;* CF_2_ ν_s_	[44]
1057	1048 (shoulder)	1048 (shoulder)	1048 (shoulder)	*α* CF_2_ ν_s_; CH_2_ w	
1076 (shoulder)	1077	1080	1080	CC ν_a_; CF_2_ w; CH_2_ w	
1148				*α* CC ν_a_; CF_2_ ν_s_	
	1170	1170	1171	CF_2_ ν_a_	[46]
1198	1198			*α* CF_2_ ν_a_; CH_2_ w	[44]
1276 (shoulder)	1276	1288	1288	CF_2_ ν_s_; CC ν_s_; CCC δ	[44,45]
1293	1293 (shoulder)			*α* CF_2_ ν_a_; CF_2_ r	[44]
1329	1330			Combination of modes, *α* theory	[46,47]
		1346/1363	1346/1363	Conformational or chemical defects	[47]
1404	1404	1402	1402	CH_2_ δ; CH_2_ w; CC ν_a_	[44]
1431	1432	1431	1432	CH_2_ δ; CH_2_ w	
2971 (shoulder)		2973	2973	CH_2_ ν_s_	[45]
2980	2976			*α* (CH_2_) ν_s_	[44]
		3010	3010	*β* CH_2_ ν_a_	[45]
3021	3015			*α* CH_2_ ν_a_	[44]

ν_s_—symmetric stretching; ν_a_—antisymmetric stretching; δ—scissoring; w—wagging; t—twisting; r—rocking; n.a.—not assigned.

### 2.3. Effect of the Polymer Type and Concentration on the Piezoelectric Module d_33_

**Figure 4:**
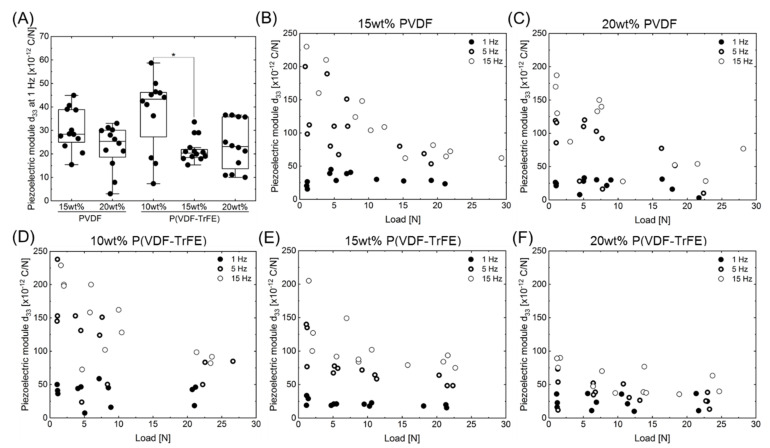
The evolution of the piezoelectric module *d*_33_ for different loads and load frequencies. (**A**) The piezoelectric module *d*_33_ at a load frequency of 1 Hz. (**B**,**C**) The piezoelectric module *d*_33_ of PVDF scaffolds produced from a 15 (**B**) and 20 wt% (**C**) solution. (**D**–**F**) The piezoelectric module *d*_33_ of P(VDF-TrFE) scaffolds produced from a 10 (**D**), 15 (**E**), and 20 wt% (**F**) solution. The 1, 5, and 15 Hz are shown as open circles with a thin line, open circles with a thick line, and filled circles, respectively. The data in (**A**) was compared using the Kruskal–Wallis test followed by a post hoc Dunn’s multiple comparison test (*p* < 0.05): boxes show 25–75% data range; line in the box—median; whiskers—1.5IQR; filled circles—the respective data; * indicates significance with *p* < 0.05 (n = 12).

Figure 4 represents the equivalent piezoelectric modulus *d*_33_ of the PVDF (Figure 4B,C) and P(VDF-TrFE) (Figure 4D–F) scaffolds calculated according to Equation (1). The measurements were conducted using the developed dynamic impact machine by analysing three scaffolds per polymer concentration for about 15 s at load frequencies of 1, 5, and 15 Hz. At each frequency, the scaffolds were subjected to the load in the range of 1–25 N. Each point in Figure 4 represents the mean value of at least 30 measured peaks that arose during the mechanical loading or unloading of the scaffold.

As can be seen, the values of the piezoelectric module are in the range of 15–250 pC/N for all scaffold types, loads, and load frequencies. The piezoelectric module increased with an increasing frequency. At higher frequencies of 5 and 15 Hz, the piezoelectric module decreased with an increasing load, whereas it remained nearly constant for a load frequency of 1 Hz, independently on the applied load. As shown in Figure 4A, the piezoelectric module *d*_33_ for 10 wt% P(VDF-TrFE) was significantly higher as compared to 15 wt% P(VDF-TrFE). No significant differences were detected within the other groups. Interestingly, the values of the piezoelectric module of the scaffolds were slightly higher for PVDF at higher frequencies than the values for P(VDF-TrFE) of the same polymer concentration. In this regard, applying a load at a frequency of 1 Hz yielded the values of the *d*_33_ module in the range from 5 to 60 pC/N. In addition, an increase in the concentration of the polymer solution, used to prepare the scaffolds, resulted in a decreased *d*_33_ module and a more compact values’ distribution for both polymer types.

### 2.4. Effect of the Polymer Type on the Zeta Potential

**Figure 5:**
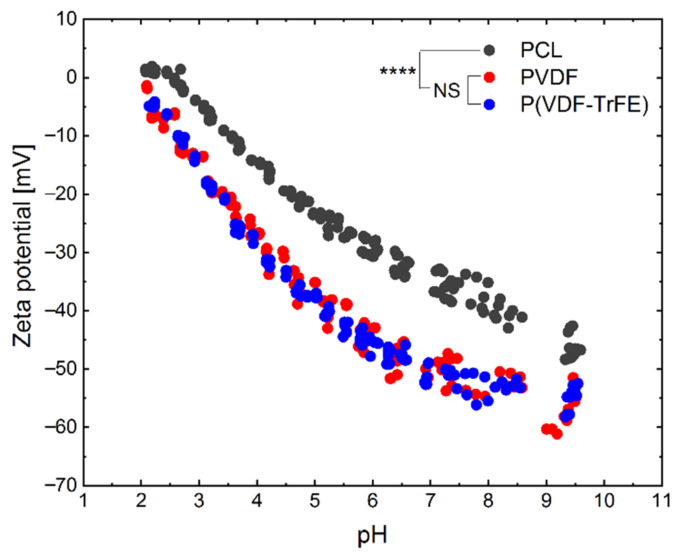
pH dependence of the zeta potentials of the 17 wt% PCL (dark grey), 20 wt% PVDF (red) and 20 wt% P(VDF-TrFE) (blue) scaffolds in a 0.01 M KCl solution. One-way ANOVA: NS—not significantly different; ****—*p* < 0.0001 (n ≥ 4).

The zeta potential of PCL, PVDF, and P(VDF-TrFE) scaffolds was examined in the pH range from 10 to 2 (Figure 5). Starting with the measurement, the zeta potential slowly increased from −55 mV, for PVDF and P(VDF-TrFE), and rose linear to higher charges at a pH below 6. Compared to PVDF and P(VDF-TrFE), the zeta potential of the PCL is higher at similar pH values. The PCL scaffolds showed a slight plateau in the acidic pH range below 2.5. The samples were showing a typical course for uncharged polymers, with the negative charge originating from adsorbed hydroxy ions [48]. The higher zeta potential values for PCL can be evoked from the less adsorption of hydroxide ions, which can be attributed to the different chemical structure and varied surface morphology. The isoelectric point (zeta potential equals 0 mV) of the PVDF and P(VDF-TrFE) scaffolds could not be detected, although it is extrapolated to be at a pH value of ≈ 2. In contrast, the isoelectric point of the PCL scaffolds was found to be at a pH ≈ 2.5. The PCL scaffolds showed the lowest zeta potential of about −46 mV in the basic pH range, whereas it was −55 mV for the PVDF and P(VDF-TrFE) scaffolds. Statistical analysis revealed no significant differences regarding the zeta potentials for PVDF and P(VDF-TrFE), whereas the PCL scaffolds presented significantly higher values (*p* < 0.0001).

### 2.5. In Vitro Biocompatibility of the Produced Scaffolds with Peripheral Glia Cells

The neonatal rat Schwann cells (nrSCs) and adult human Schwann cells (ahSCs) were cultivated on the 17 wt% PCL, 20 wt% PVDF, and 20 wt% P(VDF-TrFE) as well as on the respective control surfaces for 6 days. In order to analyse the morphology and growth pattern of the nrSCs and ahSCs, an immunofluorescence staining with a Schwann cell specific marker (α-S100) and 4′,6-diamidino-2-phenylindole (DAPI, nucleus) was performed on days 3 and 6. The lactate dehydrogenase cytotoxicity (LDH) and water-soluble tetrazolium metabolic activity assay (WST)-1 assays were used to analyse the scaffolds’ cytotoxicity and cell metabolic activity, respectively.

#### 2.5.1. nrSCs Growth on the Scaffolds

**Figure 6:**
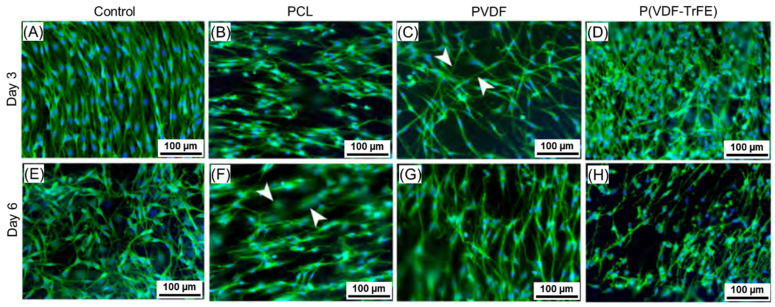
Immunofluorescence staining of the nrSCs on days 3 (**A**–**D**) and 6 (**E**–**H**) of in vitro culture. The nrSCs (green—α-S100; blue—cell nuclei, DAPI) grew evenly on the PLL-coated control surfaces (**A**,**E**) while they aligned along the fibres of the scaffolds as indicated by arrowheads in (**C**). The scaffolds were produced from 17 wt% PCL, 20 wt% PVDF, and 20 wt% P(VDF-TrFE) solutions. The arrowheads in (**F**) show exemplarily the possible cell infiltration into the fibre mats. The scale bars are 100 µm.

After three days in culture, the nrSCs were evenly distributed over the Poly-L-Lysine-coated (PLL)-coated control surfaces and the PCL, PVDF, and P(VDF-TrFE) scaffolds. Coating with PLL is a standard procedure for allowing optimal attachment and growth of nrSCs in culture [49]. Figure 6 shows the representative images of the immunofluorescence staining of nrSCs on days 3 (Figure 6A–D) and 6 (Figure 6E–H) in culture. As can be seen, nrSCs possess their typical bipolar to polygonal morphology on the PLL-coated control surfaces. The cells displayed a different morphology when cultivated on the scaffolds, where a strong bidirectional positioning with a mostly bipolar morphology could be observed for the cells cultivated on PCL (Figure 6B,F). The nrSCs cultivated on PVDF (Figure 6C,G) represented a polygonal structure with a stronger tendency to grow along the fibres of the material (Figure 6, arrowheads) with very long cytoplasmic extensions. In contrast, the nrSCs bodies on P(VDF-TrFE) appeared with a round shape, and the cells showed a polygonal morphology with shorter cytoplasmic extensions (Figure 6D,H). The morphology of the nrSCs on day 6 was similar to day 3 (Figure 6A–D vs. E–H). In some cases, the nrSCs on the PCL and PVDF scaffolds started infiltrating into the fibre mats’ porous material, as exemplarily shown with arrowheads in Figure 6F.

#### 2.5.2. ahSCs Growth on the Scaffolds

**Figure 7:**
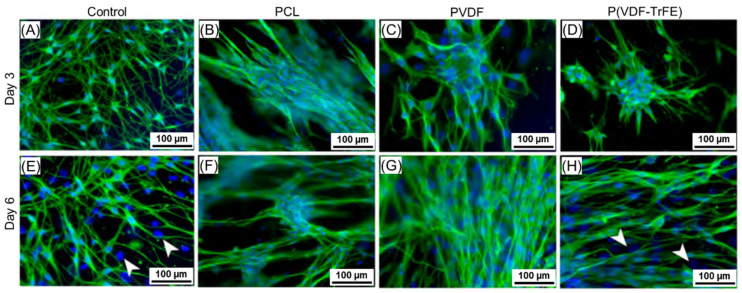
Immunofluorescence staining of ahSCs on the scaffolds on days 3 (**A**–**D**) and 6 (**E**–**H**) of in vitro culture. The ahSCs (green—α-S100; blue—cell nuclei, DAPI) grew evenly on P-ORN-laminin coated control surfaces and showed a typical polygonal to bipolar morphology. In contrast, they formed clusters of bipolar ahSCs on the scaffolds (arrowheads indicate the cells without certain α-S100 expression). The scaffolds were produced from 17 wt% PCL, 20 wt% PVDF, and 20 wt% P(VDF-TrFE) solutions. The scale bars are 100 µm.

The ahSCs were distributed uniformly after 3 days in culture on poly-L-ornithine-laminin (P-ORN-laminin) coated control surfaces and showed a typical polygonal morphology with long cytoplasmic extensions and clearly visible cell–cell contacts (Figure 7A). In contrast, they accumulated in clusters of various sizes on the scaffolds with a high density of bipolar and polygonal ahSCs. The ahSCs grown on PCL, PVDF, or P(VDF-TrFE) formed clusters. The cells formed long extensions between the clusters, which were spread across the complete cell seeding area. No specific bidirectional growth pattern was observed for the cells cultivated on all three scaffold types on day 3. In contrast, a more bidirectional growth pattern of the ahSCs was visible on day 6 (Figure 7F–H). The dominant cell type in these cultures was α-S100-immunopositive, α-S100-immunonegative cytoplasm surrounding DAPI-stained cell nuclei indicated the presence of some remaining fibroblasts (Figure 7E,H, arrowheads).

#### 2.5.3. Cytotoxicity towards Schwann Cells (SCs) and Cell Metabolic Activity of SCs

**Figure 8:**
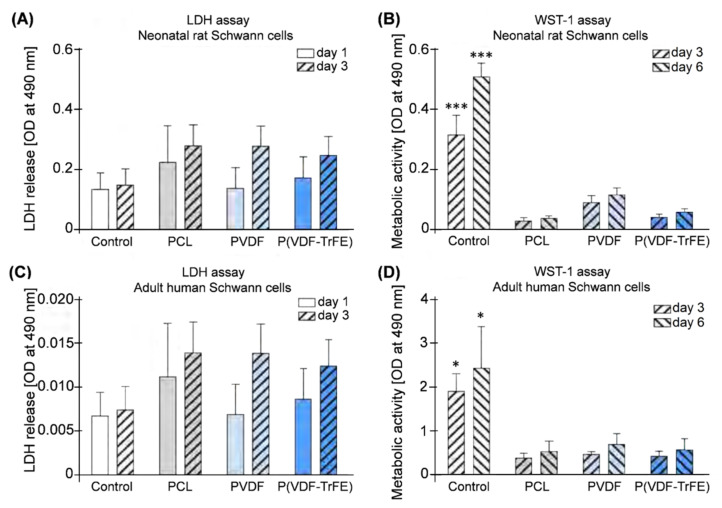
In vitro performance of 17 wt% PCL, 20 wt% PVDF, and 20 wt% P(VDF-TrFE) scaffolds with nrSCs (**A**,**B**) and ahSCs (**C**,**D**) over 6 days in culture. (**A**,**C**) LDH release measured on days 1 and 3. (**B**,**D**) Metabolic activity (WST-1 assay) of the nrSCs and ahSCs cultivated on the control surfaces and the scaffolds on days 3 and 6. The data is shown as a mean ± standard error of the mean. One-way ANOVA with Tukey’s multiple comparison test: * and *** correspond to *p* < 0.05 and *p* < 0.001, respectively (n = 3).

LDH assay was used to analyse possible toxic effects of the scaffolds, whereas WST-1 assay was performed to monitor the metabolic activity of the cells seeded onto the electrospun scaffolds. The results showed no significant differences in the released LDH between the control and the nrSCs cultured on the scaffolds (Figure 8A,C). On day 3 the levels of the released LDH were higher (but not significantly) for the scaffolds, as compared to day 1. In turn, the metabolic activity of the nrSCs cultivated on the scaffolds was significantly lower than in the control condition on days 3 and 6 (*p* < 0.001, Figure 8B). Additionally, the metabolic activity of the nrSCs cultivated on PVDF scaffolds was higher (but not significantly) compared to other scaffold types. Although the ahSCs showed lower levels of LDH release and higher levels of metabolic activity in all groups compared to nrSCs, the general pattern was similar. No significant differences in the released LDH among the scaffold types or P-ORN-laminin-coated control were detected on both days 1 and 3 in culture (Figure 8C). The metabolic activity of the ahSCs cultivated on the scaffolds was significantly lower as compared to the control group (*p* < 0.05) on days 3 and 6 (Figure 8D), but not significantly different among the scaffold types. Additionally, it should be considered that the metabolic activity of both cell types was, at all time points investigated, significantly higher in control cultures than on electrospun scaffolds. However, cells cultured on the scaffolds were viable and showed some slight increase in their metabolic activity over time as did the control cultures (Figure 8B,D).

#### 2.5.4. Qualitative Evaluation of Sensory Neurite Outgrowth

**Figure 9:**
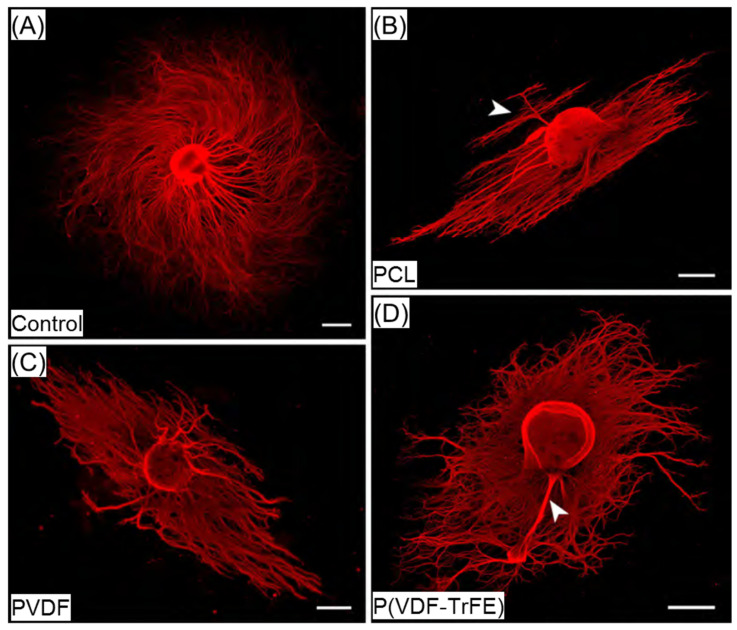
DRG neurite outgrowth on the scaffolds after 6 days of in vitro culture. (**A**–**D**) Immunofluorescence staining of dissociated DRGs (red, α-β-III-tubulin) on P-ORN-laminin coated control surface (**A**), 17 wt% PCL (**B**), 20 wt% PVDF (**C**), and 20 wt% P(VDF-TrFE) (**D**) scaffolds. Neurons of DRGs under control conditions built long neurites which grew uniformly in all directions (**A**), while neurite outgrowth on PCL displayed visible bidirectionality (**B**, arrowhead). The scale bars are 500 µm.

For detecting an effect of the piezoelectric scaffold material on neurite outgrowth from sensory neurons, dorsal root ganglia (DRGs) with mostly dissociated capsules for allowing neurite outgrowth were maintained on the PCL, PVDF, or P(VDF-TrFE) scaffolds and P-ORN-laminin coated coverslips (control) for 6 days. Since attachment of DRGs to the scaffold materials was not very stable, we did not succeed in analysing a large number of preparations. Furthermore, and especially for cultures on the scaffold materials, only few DRGs demonstrated neurite outgrowth that was robust enough for evaluation. Therefore, the results presented in this section are rather descriptive in character and do not provide any quantitative analysis of sensory neurite outgrowth behaviour. As can be seen from Figure 9, DRGs cultured on the control surfaces showed a strong neurite outgrowth with long neurites regularly distributed in a circular pattern. The neurites extending from DRGs cultured on the different scaffolds rather formed bundles (Figure 9B–D). Neurites extending from DRGs cultivated on the PCL scaffolds showed a more pronounced bidirectional outgrowth pattern along the fibres of the material. We even detected a bundle of neurites that started to grow against that direction, but in some distance from the DRG, the neurites still orientated along the fibres (Figure 9B, arrowhead).

When cultured on PVDF, the neurites extending from the DRGs were oriented along the fibres of the scaffolds (Figure 9C). As compared to DRGs cultured on PCL, the neurite bundles seemed slightly larger in diameter, while their bidirectional outgrowth pattern was less obvious. In contrast to DRGs cultured on PCL and PVDF, the neurite outgrowth on P(VDF-TrFE) showed no distinct pattern and, in comparison to the plain-coated-surface control, also no even distribution (Figure 9D). In some cases, DRGs were initially not dissected completely from their nerve fibres, which then resulted in the formation of large neurites bundles (Figure 9B,D, arrowheads). Any specific support of neurite distance outgrowth by either scaffold was not detectable from our cultures.

## 3. Discussion

Biosynthetic nerve conduits can be considered as potential candidates to replace the gold standard, the autologous nerve grafts, to repair PNIs. Although a variety of materials and their regeneration enhancing properties are known, developing a conduit possessing optimal characteristics is still a vivid field [49,50]. While the application of PCL, an FDA approved polymer, has shown great promise in rat sciatic nerve injury models for peripheral nerve regeneration [14,50,51], the results of our own in vivo study revealed that the application of our electrospun PCL scaffolds induced massive foreign body response and thus impaired the axonal regeneration after 13 weeks post-transplantation into sciatic nerve gaps of Wistar rats [15]. Alternatively, Lee et al. demonstrated that electrospun scaffolds made of P(VDF-TrFE), a polymer possessing piezoelectric activity, could enhance the axon regeneration and thus bear a huge potential for spinal cord repair [30]. Thus, PVDF-based materials providing electrical stimuli to the cells can be considered as prospective candidates to engineer artificial nerve conduits [52]. Although their biocompatibility and regeneration promoting properties have already been proven [53,54], there is, to our knowledge, still no single-lab comparative study of PVDF and P(VDF-TrFE) electrospun scaffolds in terms of structural properties, piezoelectric activity and in vitro biocompatibility. In this regard, the current work aimed mainly at evaluating the effect of the electrospinning of PVDF and P(VDF-TrFE) solutions on the structural properties, surface charge and piezoelectric module *d*_33_ of the electrospun scaffolds. Moreover, we reveal whether PVDF, a cheaper alternative to costly P(VDF-TrFE), could perform better than its copolymer in terms of in vitro biocompatibility with peripheral glia cells. For this, the rat and human Schwann cells were cultured on the electrospun PVDF and P(VDF-TrFE) scaffolds. Along with the morphological (immunofluorescence staining) and biochemical (LDH and WST-1 assays) analysis of the response of SCs to electrospun scaffolds, we evaluated the material-associated neurite outgrowth from sensory DRG neurons. For in vitro studies, a non-piezoelectric PCL was used as control.

### 3.1. Electrospun PVDF Scaffolds Are Similar to P(VDF-TrFE) Ones in Terms of the Structural Properties and Piezoelectric Activity

The most important scaffold’s parameters that define its piezoelectricity response are the crystallinity degree of the piezoelectric material and the content of the polarised β-phase in the crystal lattice. As expected, the analysis of the scaffolds’ morphology using SEM revealed an increase in the fibre diameter with an increasing polymer concentration. Although the rotational speed of the drum collector was set to minimal in order to obtain the electrospun scaffolds without fibre alignment, the fibre alignment can be observed in SEM images of 20 wt% P(VDF-TrFE). The fibre diameter of the produced scaffolds was found to be in the range of 1–2 µm, with a smaller fibre formation in the scaffolds produced from 10 wt% P(VDF-TrFE). In order to analyse the effect of the electrospinning process on the content of the crystalline β-phase, Raman microscopy was conducted for the raw materials and the produced 20 wt% PVDF and P(VDF-TrFE) scaffolds. In general, the phase transformation within the PVDF takes place upon applying external cures, such as the mechanical drawing, electrical poling and heat-treatment [24,35], shearing [55], compression [38,56], and others. On the other hand, the copolymerisation of VDF with TrFE into P(VDF-TrFE) has been shown to result in materials with a high β-phase content. The polymerisation ratio can easily be tailored to produce P(VDF-TrFE) materials with the desired β-phase content and the respective piezoelectric properties [35].

In regard to the processability of the P(VDF-TrFE) and PVDF solutions, the latter is rather difficult to electrospin due to its higher viscosity compared to P(VDF-TrFE). During the electrospinning process, electrical poling and shearing occur due to the application of the electric field, ejection of a fibre from the tip of the needle, and its subsequent transportation to the collector. These processes yield in the orientation of dipole moments and phase transformation. In our previous work [38], we evaluated the crystallinity and β-phase content of PVDF after electrospinning using the Fourier-transform infrared spectroscopy (FTIR) and differential scanning calorimetry (DSC). The results indicated that electrospun PVDF fibre mats possessed a higher crystallinity (45% vs. 55% for raw and electrospun PVDF, respectively) and increased content of the β-phase, as compared to the raw PVDF. The results of this work corroborate our previous findings as well as the available information in the literature: the β/α ratio increases from 0.4 for the raw PVDF to 2.3 after the electrospinning. By comparing the Raman and FTIR spectra as well as correlating the quantified FTIR data with the β/α ratio calculation based upon the intensities of the respective Raman bands of PVDF at 839 and 794 cm^−1^ (this work as well), Xu et al. revealed the β/α ratio of around 2 yielding more than 90% of the β-phase content in the sheared and temperature treated PVDF films [55]. Our results of the Gaussian deconvolution of the Raman bands in the wavenumber range of 740–930 cm^−1^ confirmed the hypothesis that the electrospinning does not affect the β-phase content within P(VDF-TrFE), which could be associated with the absence of the α-phase.

Based on the results of the Raman microscopy, it could be hypothesised that the α-phase undergoes a noticeable transformation to β-phase upon electrospinning. In support of this hypothesis, we observed no significant difference in the zeta potential among PVDF and P(VDF-TrFE) electrospun fibre mats. This correlates to the changes in surface charge of the materials within a solution with different pH values. The knowledge of the zeta potential helps to optimise surface modification processes to develop suitable scaffolds for cell growth [57]. In neural regenerative medicine, the properties of the zeta potential and the spatial conformation of the polymers can influence the cell behaviour in response to electrical stimulation [58]. In this regard, our results demonstrate the isoelectric point (IEP) of the scaffolds to be ≈2.5 for PCL and at ≈2 for PVDF and P(VDF-TrFE), which possess a negatively charged surface due to the absorption of hydroxy ions [48]. In addition, the IEP could shift to lower values when acidic surface groups are present [48,57]. Castaño et al. measured a negative zeta potential of −52.7 mV at a physiological pH and an IEP of 3.0 for electrospun PCL scaffolds [59], which is in accordance with our findings. At a physiological pH, our results for PCL show a zeta potential of approximately −37 mV. This difference to Castaño et al. could be explained by a different topography of the fibres, resulting from different electric field strength during electrospinning [57]. The greater surface roughness could have raised the zeta potential [60]. For the PVDF and PVDF-TrFE nanofiber scaffolds, the zeta potential was measured to be approximately −51 and −54 mV at the physiological pH, respectively [61]. It was revealed that the surface charge of the piezoelectric materials remains negative above the pH value of 3, which correlates to our results, showing a value of approximately −50 mV at the physiological pH.

In order to compare the piezoelectric activity of the produced scaffolds, a dynamic impact load machine was developed, validated, and used to measure the voltage generated by the electrospun scaffolds under a range of loads and load frequencies. The developed approach yields excellent results for a load frequency of 1 Hz, where the calculated piezoelectric module of 15–60 pC/N is independent of the applied loads and correlates with the information within the literature for PVDF and P(VDF-TrFE) films and electrospun scaffolds. Guo et al. revealed the piezoelectric module of 24.9 ± 2.9 pC/N for PVDF electrospun scaffolds with randomly oriented fibres [62]. In turn, Wu et al. found that an increase in the alignment of the PVDF fibres in electrospun scaffolds results in an increase in the piezoelectric module *d*_33_ from 16.8 ± 1.4 (randomly oriented fibres) to 27.4 ± 1.5 (aligned fibres) [63]. The incorporation of multiwall carbon nanotubes into PVDF yielded a further increase in *d*_33_ to 31.3 ± 2.1 pC/N for the scaffolds with aligned fibres. An increase in *d*_33_ upon increasing the load frequency from 1 to 5 and 15 Hz was detected. This could be attributed to the measurement of the generated voltage outside the viscoelastic range of the scaffolds. During the application of the load, the fibres were prevented from expanding laterally by the electrodes. This could result in the generation of mechanical stresses perpendicular to the actual load and storage of the induced charge.

### 3.2. PVDF and P(VDF-TrFE) Electrospun Scaffolds Are Biocompatible with Schwann Cells and Neurite Outgrowth In Vitro

Schwann cells were chosen to evaluate their response to the electrospun scaffolds in vitro to mimic a typical environment after a peripheral nerve injury, as they account for 90% of nucleated cells within the peripheral nerves [64] and are crucial for the nerve function and repair. While primary nrSCs are easy to cultivate in vitro for evaluating the biocompatibility of engineered scaffolds for the peripheral nerve regeneration, use of adult human SC represents a more challenging but translational approach towards future clinical application in patients [65]. The immunofluorescence analysis of the ahSCs and nrSCs cultivated on the PCL, PVDF, or P(VDF-TrFE) scaffolds revealed no distinct differences in the cell growth patterns among the scaffold types. The nrSC cultivated on the scaffolds showed a cell specific bipolar or polygonal morphology, although it differed as compared to those cultures maintained on plain, but PLL-coated, surfaces. In turn, the ahSCs cultivated on the electrospun scaffolds displayed a bidirectional growing pattern on the PCL scaffolds. In contrast, they did not exhibit a certain alignment along the PVDF or P(VDF-TrFE) fibres, which could be attributed to a different fibre alignment of the produced scaffolds. It is well known that the scaffold morphology (fibre size, fibre alignment, porosity) and structural properties (crystallinity, wettability) influence the cell morphology, growth, and differentiation [66,67]. Lins et al. analysed the effect of the PVDF fibre alignment on the behaviour of undifferentiated monkey neural stem cells (NSCs), and the NSCs differentiated into neuronal and glial cells [68]. The authors revealed that the growth pattern of undifferentiated stem cells and glial cells was not affected by the fibre alignment, whereas the stem cells differentiated towards neuronal cells possessed an elongated morphology on the aligned fibres. In addition, the aligned fibres improved the formation of bands of Büngner by Schwann cells and enhanced a directed neurite outgrowth [69,70,71] as well as cell migration, which plays an essential role within the nerve regeneration after PNI [72,73]. In this regard, our study results do not allow for drawing conclusions on scaffold material effects on the migratory behaviour of either Schwann cell type. Migration of repair Schwann cells into a novel type of NGCs, however, is largely accepted to be beneficial for a successful regeneration process [74].

As confirmed by using LDH assay, PCL, PVDF, and P(VDF-TrFE) electrospun scaffolds did not exhibit a cytotoxic effect on the nrSCs and ahSCs, as compared to the cells seeded on the PLL-coated control surfaces. The biochemical analysis showed that the general pattern of LDH release of ahSCs was similar to that of nrSCs. Nevertheless, the total amount of the released LDH was considerably lower and the metabolic activity higher for ahSCs on the control surfaces compared to the nrSCs. This could be attributed to cell type specific differences in metabolism and LDH release. The metabolic activity of cells analysed using WST-1 assay over a time period of 6 days was significantly reduced for the cells cultivated on the electrospun scaffolds, as compared to the cells on the plain, but coated, control surfaces. The metabolic activity of the nrSCs and ahSCs cultivated on the PVDF scaffolds was higher (but not significantly) compared to the cells on the PCL and P(VDF-TrFE) scaffolds. It is noteworthy that a reduction of metabolic activity from control conditions to cultures on electrospun scaffolds, is not necessarily an indication of a reduced cell viability. We did not see impaired cell viability of cells successfully attached to the electrospun scaffolds, when analysing our cultures in immunofluorescence. We have demonstrated a similar phenomenon with another biomaterial before [11]. A significantly higher metabolic activity measured under control conditions has rather to be attributed to the highly optimised culture conditions induced by the cell-specific coating of plain cell culture surfaces [11].

The qualitative monitoring of the neurite outgrowth from sensory DRG neurons cultivated on PCL, PVDF, and P(VDF-TrFE) did not reveal any differences in distance outgrowth. It could be hypothesised that in the case of static cultivation, the scaffold morphology and fibre orientation play a prominent role in controlling the neurite outgrowth. In this regard, Lee et al. revealed that the neurites formed by DRG neurons on PVDF and P(VDF-TrFE) scaffolds and cultivated for 4 days were the longest (around 2 mm) for aligned and annealed P(VDF-TrFE) electrospun scaffolds [33]. In turn, no detectable differences with regard to neurite length in our study could be attributed to a lower fibre alignment rate. Wu et al. demonstrated that the co-culturing of SCs with DRGs on P(VDF-TrFE) scaffolds promoted a longer neurite extension compared to the scaffolds without SCs [32]. The piezoelectric effect combined with an optimal fibre alignment can improve a directed neurite extension. Nevertheless, the intrinsic piezoelectric effect of P(VDF-TrFE) due to the presence of the TrFE copolymer failed to provide a detectable advantage over PVDF for neurite outgrowth.

## 4. Concluding Remarks

To our knowledge, the present study deals, for the first time, with a comparative analysis of PVDF and P(VDF-TrFE) electrospun scaffolds regarding their morphological and structural properties, piezoelectric activity as well as their biocompatibility to peripheral nerve cells in vitro. We revealed a sufficient scaffold biocompatibility with Schwann cells and no apparent cytotoxicity regarding neonatal rat and adult human Schwann cells. With regard to the guided regrowth of axons, the PVDF scaffolds were able to enhance their bidirectional outgrowth. A similar in vitro performance of the PVDF and P(VDF-TrFE) scaffolds produced using electrospinning could be attributed to their comparable morphology and the content of the piezoelectric crystalline β-phase as well as a similar surface charge. The PVDF scaffolds represent a cheaper alternative to P(VDF-TrFE) and a high flexibility in adjusting the β-phase content in the course of the electrospinning. Along with an easier processability and control over the β-phase content in P(VDF-TrFE) by adjusting the polymerisation degree, both PVDF and P(VDF-TrFE) scaffolds represent a piezoelectric activity required for the electrical stimulation of a damaged nerve. Thus, these scaffolds can be suggested as promising candidates for future development of artificial nerve conduits for the peripheral nerve injury repair and regeneration.

## 5. Materials and Methods

Unless stated otherwise, the materials were purchased from Carl Roth GmbH + Co. KG (Karlsruhe, Germany), and the volume concentration of a solution is expressed as % *v*/*v*. PVDF (Sigma-Aldrich Chemie GmbH, Taufkirchen, Germany) with a molecular weight of ~530 kDa in pellet form and P(VDF-TrFE) 70/30 mol% (Piezotech Arkema Group, Pierre-Benite, France) with a molecular weight of ~695 kDa in powder form were dissolved in a mixed solution of two solvents; N,N-dimethylformamide (DMF, Fluka Analytical, Seelze, Germany) and acetone (AppliChem GmbH, Darmstadt, Germany) in a weight ratio of 6:4. For the identification of favourable conditions for producing the scaffolds, concentrations of 10, 15, and 20 wt% PVDF and 10, 15, and 20 wt% P(VDF-TrFE) were prepared at 50 °C and stored at room temperature overnight prior to electrospinning. As a non-piezoelectric material, 17 wt% polycaprolactone (PCL, Sigma-Aldrich Chemie GmbH, Taufkirchen, Germany) in pellet form with a molecular weight of ~80 kDa was dissolved in 2,2,2-Trifluorethanol (TFE, abcr GmbH, Karlsruhe, Germany) for the scaffold production via electrospinning.

### 5.1. Preparation of the Scaffolds Using Electrospinning

Utilising the electrospinning technique, the polymer solution was transferred into a 10 mL syringe with a Luer-lock tip and an orthogonally cut-ended needle (all from B. Braun Melsungen AG, Melsungen, Germany). A syringe pump (Kd Scientific Inc., Holliston, MA, USA) was used to control the solution flow rate. A preliminary optimisation of the electrospinning process parameters was conducted to produce PCL, PVDF and P(VDF-TrFE) scaffolds with comparable fibre diameters. This included the application of a high voltage in the range of 10–30 kV between the syringe needle and a rotating drum collector (rotation speed of 500–1000 rpm). The fibres were collected on a grounded piece of aluminium foil (6 × 20 cm^2^) which was placed on the cylindrical drum collector (diameter of 15 cm, width of 12 cm) at a distance of 16–26 cm from the tip of the needle. The electrospinning time of each sample was adjusted to obtain the scaffolds of the comparable thickness (around 100 µm) under a temperature range of 22.0–25.5 °C and a humidity between 33 and 50%. Table 3 shows the optimal parameters applied for the production of PCL, PVDF, and P(VDF-TrFE) electrospun scaffolds.

### 5.2. Morphological Characterisation

The morphology of the PVDF (10, 15, 20 wt%) and P(VDF-TrFE) (10, 15, 20 wt%) electrospun scaffolds was characterised for the fibre diameter and fibre mat thickness. For this, the scaffolds were cut and mounted onto sample holders with a double-sided adhesive conductive carbon tape. Before observation, the samples were sputter-coated with gold-palladium in an EMITECH SC7620 sputter coater (Quorum Technologies Ltd., Lewes, UK) under vacuum. The samples were then imaged using the scanning electron microscope (SEM, VP-SEM S-3400 Type II, Hitachi High-Technologies Europe GmbH, Krefeld, Germany). The images were taken at a magnification of 2500× (fibre diameter) and 500× (fibre mat thickness), an accelerating voltage of 15 kV and a working distance of 10 mm. For the thickness measurements, the scaffold samples produced from 17 wt% PCL, 20 wt% PVDF, and 20 wt% P(VDF-TrFE) were cut in liquid nitrogen to get a clear-cut edge without compressive damage, mounted in vertical sample holders with the cut edge facing upwards and imaged as described above. The fibre diameter was analysed based on at least three randomly taken images and measured manually. In total, 180–300 and 50 measurements were taken to analyse the fibre diameter and thickness, respectively.

### 5.3. Development and Validation of the Modified Dynamic Impact Load Machine

**Figure 10:**
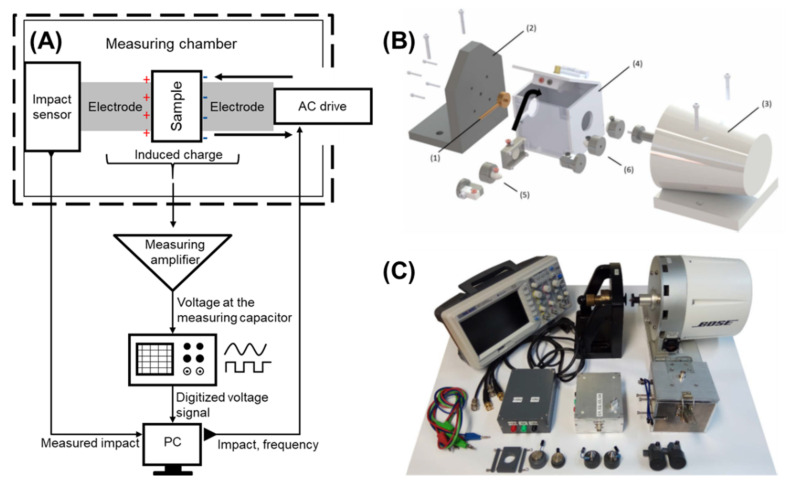
Representation of the developed dynamic impact loading machine. (**A**) Schematic representation of the dynamic measurement method. (**B**) CAD drawings of the structure of the measurement chamber: (1) load cell, (2) mounting bracket, (3) BOSE solenoid drive, (4) measurement chamber, (5) first electrodes with the sample holders (left—*d*_31_/*d*_32_; centre and right—*d*_33_), and (6) moving electrodes (left—*d*_31_/*d*_32_; centre and right—*d*_33_). (**C**) A photograph showing an overview of the main parts used in the dynamic measurement method.

The dynamic impact load machine was developed to analyse the piezoelectric activity of the produced PVDF and P(VDF-TrFE) scaffolds. The main working principle of the developed dynamic impact machine is schematically shown in Figure 10A.

The developed method consists of a measurement chamber, the magnetic coil drive BOSE Electroforce LM1 TestBench with a calibrated 45 N load sensor (TA instruments Inc., New Castle, UK), a measurement amplifier, the acquisition unit, processing and digitalisation software of the measurement data. The measurement chamber was designed with a metal housing on all sides (Figure 10B (4)) to shield the interior from the electrical and magnetic interfering fields. A photograph of the parts of the dynamic load system is shown in Figure 10C. The mechanical loading was controlled using the WinTest7 software (TA Instruments Inc., New Castle, UK). The generated voltage was measured through an amplifier using a Siglent SDS1102CML+ oscilloscope (Siglent Technologies Germany GmbH, Augsburg, Germany). The oscilloscope signal was acquired using the EasyScopeX software (Siglent Technologies Germany GmbH, Augsburg, Germany). A specific software was developed with Visual Basic in a Microsoft Excel environment to process the generated voltage, applied mechanical load, and load frequency.

The validation of the developed measurement amplifier was conducted using a lead-zirconate-titanate (LZT, PI Ceramic GmbH, Lederhose, Germany) piezo-ceramic plate (diameter of 15 mm, thickness of 1 mm, Figure S12) possessing an electrical resistance of 500 Ω and a capacitance of around 2200 pF. The electric charge was measured by the amplifier and compared with the induced charge measured directly from the LZT piezo-ceramic plate. For these measurements, the LZT ceramics were preloaded with 0.5 N. The final loading with 0.25 N was applied over a load frequency range of 1–50 Hz. Both measurement methods showed the same charge development over the increasing load frequency (Figure S13A). In a study regarding the relationship between the LZT piezoelectric module and the mechanical load at a constant load frequency of 20 Hz, the LZT ceramics showed a constant piezoelectric module of 60 × 10^−9^ C/N over a load magnitude up to 2 N. Above 2 N the *d*_33_ module decreased with an increasing load magnitude at a constant load frequency. At a constant load of 0.5 N, the generated charge remained constant over the load frequencies above 14.5 Hz. Below this load frequency, a fast discharge resulted in only partial detection of the induced charge (Figure S13B). Further, a cellulose sample (Rotilabo^®^-Rundfilter, Ø 15 mm, Mercateo AG, Munich, Germany) of the same size was selected as a non-piezoelectric material and measured under the same conditions as the LZT. No charge formation on the surface was detected, independently of the load magnitude and the load frequency for the cellulose sample. A more detailed overview of the development and validation stages, and the respective components of the dynamic impact load machine can be found in the Supplementary Materials (Figures S5–S14, Equations (S1)–(S12)).

### 5.4. Measurement of the Piezoelectric Module d_33_

**Figure 11:**
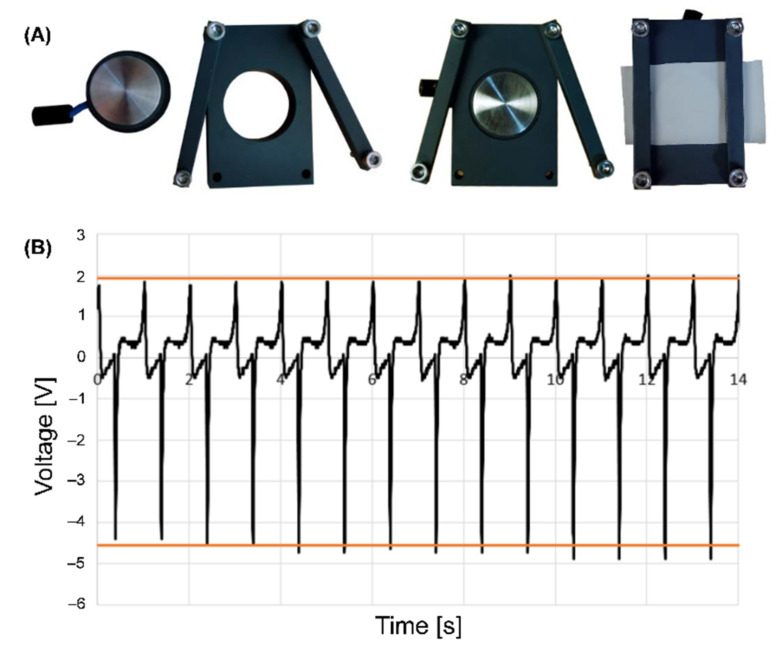
Representative images of the set-up of the electrode system with the scaffolds (**A**) and voltage profile over the time monitored with an oscilloscope (**B**).

In order to analyse the direct piezoelectric effect of the PVDF and P(VDF-TrFE), the scaffolds with sizes of 6 × 4 cm^2^ were fixed on the static electrode, as shown in Figure 11A. Afterwards, the mechanical load was applied using a moving electrode. The generated voltage was measured and recorded using the developed software. Figure 11B shows the representative dependence of the generated voltage over the measuring time. The influence of the load magnitude and frequency on the piezoelectric module was investigated. The direct piezoelectric module *d*_33_ was calculated according to Equation (1):

(1) $$d_{33}=\frac{U\cdot C}{F}$$

where *U* is the generated voltage [V], *C* is the capacitance of the used capacitor [F], and *F* is the applied load [N]. The capacitor C can be selected based upon the applied frequency. According to the validation results, the capacitor with a capacitance of 100 pF was used to calculate the *d*_33_ at 1, 5, and 15 Hz.

### 5.5. Raman Microscopy

To analyse the effect of the electrospinning process on the structural properties and phase composition of the produced fibre mats, Raman microscopy of the raw materials and the fibre mats made from 20 wt% PVDF and 20 wt% P(VDF-TrFE) solutions was conducted. In brief, the samples were placed on a glass slide using an adhesive tape and imaged with an alpha300RA microscope (WiTEC GmbH, Ulm, Germany). Before each measurement, a Si wafer was utilised to calibrate the system with the Si peak at 520 cm^−1^, which corresponds to the crystalline Si-Si bond of longitudinal optical phonon vibrations. A green line (532 nm excitation light) from a diode Nd-YAG laser was focused onto the samples using a 100×1 objective (EC Epiplan-Neofluar DIC, numerical aperture 0.9, Carl Zeiss AG, Jena, Germany). The spectra were collected with an Ultra High Throughput Spectrometer (UHTS300) with a grating of 600 grooves/mm and a Peltier-cooled CCD camera (spectral centre at 2150 cm^−1^). The microscope and spectra acquisition were controlled using the Control FIVE.plus software (WiTEC GmbH, Ulm, Germany). The spectra were collected in the wavenumber range of 200–3750 cm^−1^ using a laser power of 25 mW, 5 s integration time, and a total number of 100 accumulations. The obtained spectra were analysed using the Project FIVE.plus software (WiTEC GmbH, Ulm, Germany). For the analysis, a cosmic-ray removal (filter size 2, dynamic factor 2), smoothing using a Savitzky-Golay (points 3, polynomial order 2), and background substation with a shape function (shape size 250, noise factor 3) were performed. The intensity values were normalised using the following Equation (2):

(2) $$I_{norm}=\frac{I_{y}-I_{min}}{I_{max}-I_{min}}$$

where *I_norm_* is the normalised intensity, *I_max_* is the maximum intensity value, *I_min_* is the minimum intensity value, and *I_y_* is the measured intensity [arb. un.].

Six spectra of the raw materials (PVDF pellet and P(VDF-TrFE) powder) and produced fibre mats were collected and processed as described above. A peak deconvolution in the range of 740–930 cm^−1^ was performed in OriginPro 2021 (OriginLab Corporation, Northampton, Massachusetts, United States) utilising a Gaussian distribution in order to calculate the transformation of the amorphous α- to crystalline β-phase during the electrospinning process for PVDF as well as the effect of the electrospinning on P(VDF-TrFE) materials. The calculation of the β/α ratio for the PVDF before and after the electrospinning was performed based on the intensity of the bands at 797 (α-phase) and 839 cm^−1^ (β-phase) according to the following Equation (3) [75]:

(3) $$I_{\frac{\beta }{\alpha }}=\frac{I_{\beta (839)}}{I_{\alpha (797)}}$$

where *I*_β_ and *I_α_* are the intensities of the bands at 839 and 797 cm^−1^ [arb. un.], respectively.

In turn, the calculation of an integral area of the peaks corresponding to the α- and β-phases of the PVDF and P(VDF-TrFE) was conducted using the following Equation (4):

(4) $$A_{p}[\%]=\frac{A_{p}}{A_{t(740-930)}}\times 100\%$$

where *A_p_* is the area of the respective bands (797/839 cm^−1^ for PVDF and 845 cm^−1^ for P(VDF-TrFE)), and *A_t_* is the total area under the curve in the region of 740–940 cm^−1^ [arb. un.].

### 5.6. Zeta Potential Measurements

Electrospun scaffolds of 20 wt% PVDF, 20 wt% PVDF, and 17 wt% PCL were chosen to determine the surface zeta potential by using the analyser SurPASS 3 (Anton Paar GmbH, Graz, Austria). The zeta potential ζ was calculated in accordance to the Helmholtz–Smoluchowski according to the following Equation (5):

(5) $$\zeta =\frac{\Delta U_{str}}{\Delta p}\cdot \frac{\eta }{\varepsilon \cdot \varepsilon_{0}}\cdot \kappa_{B}$$

where *ζ* is the zeta potential [V], Δ*U_str_* is the streaming potential [V], Δ*p* is the differential pressure [Pa], *κ_B_* is the electrolyte conductivity [Ω/m], *η* is the electrolyte viscosity [Pas], *ε* is the dielectric coefficient of the electrolyte, and *ε*_0_ is the permittivity [A⋅s⋅V/m].

For each measurement, two samples with the dimensions of 1 × 2 cm^2^ were used. The zeta potential measurements were repeated five times for each scaffold type. A 0.001 M potassium chloride solution was used as the electrolyte solution, whereas a pressure difference of 200–600 mbar was applied to generate the streaming and the values were calculated in the range of 200–500 mbar. In some cases, the pressure difference had to be reduced to ensure the linear correlation of Δ*U_str_*/Δ*p*. Three measurements per sample were obtained in a pH range of 10 to 2.

### 5.7. In Vitro Evaluation of the Produced Electrospun Scaffolds

#### 5.7.1. Cell Cultivation

Neonatal rat Schwann cells (nrSCs) were harvested from sciatic nerves isolated from Hannover Wistar rat pups from postnatal days 1–3 (passages 1–3) and collected in Hank’s balanced salt solution (HBSS, GIBCO, Thermo Fisher Scientific, Waltham, MA, USA) with 1% penicillin/streptomycin (Pen/Strep, GIBCO, Thermo Fisher Scientific, Waltham, MA, USA) and 1% amphotericin B (Biochrom GmbH, Berlin, Germany). To dissociate the cells enzymatically, the sciatic nerves were incubated in a dissociation solution for 45 min at 37 °C. The dissociation solution contained high glucose Dulbecco’s modified Eagle medium (DMEM, GIBCO, Thermo Fisher Scientific, Waltham, MA, USA) supplemented with 0.04% foetal calf serum (FCS, GIBCO, Thermo Fisher Scientific, Waltham, MA, USA), 0.004% Pen/Strep, 160 U/mg collagenase IV (Worthington Biochemical Corporation, Troisdorf, Germany), and 0.05% dispase II protease. Afterwards, the tissue was mechanically dissociated and processed according to our previously published protocol [76]. Primary cells were purified by immunopanning with magnetic Pan mouse IgG Dynabeads^®^ (Dynal Biotech ASA, Oslo, Norway) linked to an anti-Thy1-antibody (own production from hybridoma cell line) directed against surface glycoproteins of fibroblasts according to [76]. The purification was repeated 2–3 times until the cultures reached at least 90% purity [11]. The purified cells used for the experiments were cultured in nrSC medium in PLL-coated sterile culture flasks with a change of medium every 2–3 days, followed by splitting before reaching 90% confluency.

Adult human Schwann cells (ahSCs) were obtained from residues of peripheral nerve transplants harvested during reconstructive surgery in vivo from male or female donors aged 13–60 years [77]. The ahSCs were dissociated and cultivated as described before [78]. The purified cells were cultured in poly-L-ornithine-laminin (P-ORN-laminin) coated culture flasks in a specific ahSC culture medium in 1% BSA at 37 °C and 5% CO_2_. The ahSCs culture medium consisted of melanocyte growth medium (PromoCell, Heidelberg, Germany) supplemented with 2 µM forskolin, 1% Pen/Strep, 1% amphotericin B, 10 ng/mL human fibroblast growth factor (PeproTech EC Ltd., London, UK), 5 g/mL bovine pituitary extract (BPE-26, PromoCell, Heidelberg, Germany), 2.5 ng/mL insulin (GIBCO, Thermo Fisher Scientific, Waltham, MA, USA), and 10 nM recombinant heregulin (rHRG, R&D Systems GmbH, Wiesbaden, Germany). After 24 h, the medium was changed to serum-free ahSC culture medium. The ahSCs were cultivated until 90% confluency was reached, or the cells were used immediately for experiments.

#### 5.7.2. Cell Seeding onto the Scaffolds

To evaluate the cell response to the electrospun scaffolds, the Schwann cells (nrSC and ahSC) were seeded on top of the PCL, PVDF, and P(VDF-TrFE) scaffolds. Before usage, the scaffolds were cut with the help of circular cover slips (MENZEL Cover Slips, 24 × 24 mm^2^, Gerhard Menzel GmbH, Braunschweig, Germany) and a scalpel (Feather ^®^ disposable scalpel (No. 15), Fisher Scientific, Schwerte, Germany). Afterwards the scaffolds were placed in 24-well plates, washed twice with sodium chloride 0.9% solution (B. Braun Melsungen AG, Melsungen, Germany) for approximately 1.5 h, fixed with the pressure of Cell Crowns^TM^ (CellCrowns^TM^24, Scaffdex Oy, Tampere, Finland) and subsequently air-dried for 24 h. As a control, culture plate wells and coverslips were coated with PLL or P-ORN-laminin for nrSCs and ahSCs, respectively. In three sister cultures, 5 × 10^4^ cells were seeded in 50 µL drops of cell specific culture medium placed in the middle of the scaffolds and preincubated for 60 min under cell type specific conditions as described above. To prevent flushing of the cells to the well bottom and drying up, first 50 μL and then 100 µL of medium was added after 20 min each. After 4 h of incubation, the wells with the scaffolds were filled up to a total volume of 1 mL of cell specific culture medium. Afterwards, the cells were cultivated for 3 and/or 6 days depending on the assay used. In the experiments with ahSC, the medium was changed on day 1 from attachment to culture medium after removing supernatant for the lactate dehydrogenase assay. If the cells were incubated up to day 6, the total volume in each well was increased to 1.5 mL on day 3. For the biochemical cytotoxicity analysis, an additional well was filled with pure culture medium as a negative control. The experiments were repeated twice with cells from different preparations and passages (passages 7–11 for nrSCs and 3–8 for ahSCs).

To examine the cell response of the scaffolds, two different biochemical methods were used: the LDH cytotoxicity assay (Pierce Biotechnology Inc., Rockford, IL, USA) and the WST-1 metabolic activity assay (Sigma-Aldrich Chemie GmbH, Taufkirchen, Germany). Both assays were conducted three times (n = 3) with two sister cultures per condition.

#### 5.7.3. Lactate Dehydrogenase Assay (LDH)

Damaged cells or cells treated with toxic substances release the cytosolic lactate dehydrogenase, which is an indicator for the state of cells and tissues, whereas the amount of released LDH is proportional to the level of cytotoxicity. The LDH assay was performed on days 1 and 3. For this, 75 µL of the supernatant from each well was transferred into a 1.5 mL tube (Sarstedt AG & Co. KG, Nümbrecht, Germany) and centrifuged at 10,000 rcf for 15 min at 0 °C. From each tube, 50 µL of supernatant was transferred into a 96-well plate (Thermo Fisher Scientific, Waltham, MA, USA) together with a duplicate of positive control provided with the kit. Each sample was incubated with the LDH substrate mixture, which was prepared according to the provided protocol. After 30 min, the reaction was stopped with a stop solution and the optical density (OD) of each sample was measured at 490 nm with a multi-well plate reader ELX800 (BioTek Instruments Inc., Winooski, VT, USA).

A preliminary test was performed to ensure that the LDH assay is sensitive for the desired cell number. For this purpose, the nrSCs were seeded in two sister cultures in a 24-well plate with 1 mL of nrSC medium in different cell numbers (0.25 × 10^5^, 0.5 × 10^5^, 1 × 10^5^, 2 × 10^5^, 4 × 10^5^ cells) and treated for 72 h with 0.1 µM staurosporine. Staurosporine is a protein kinase inhibitor, which induces apoptosis and creates an artificial toxic environment for the cells [79]. Untreated controls (positive control) for each cell number and wells with culture medium (negative control) were added as a reference. The LDH assay was performed every 24 h as described above and repeated three times.

#### 5.7.4. Water-Soluble Tetrazolium Assay (WST-1)

The metabolic activity of cells was evaluated by means of the WST-1 cell proliferation reagent. To perform the WST-1 assay, the supernatant in each well was removed and the scaffolds transferred to new wells on day 3 and 6. Additionally, a well was filled with cell specific culture medium as a negative control. Then, 400 (ahSCs) or 500 µL (nrSCs) of cell specific culture medium containing the WST-1 compound (1:10) were added to the wells and incubated for 3.5 h at 37 °C in a humidified atmosphere with 5% (ahSCs) or 8% (nrSCs) CO_2_. Subsequently, 100 µL in triplicates were transferred to a 96-well plate, and the OD was measured at 450 nm using a multi-well plate reader.

### 5.8. Neurite Outgrowth from Primary Dissociated Rat Dorsal Root Ganglia

#### 5.8.1. Preparation and Cultivation of DRGs

Dorsal root ganglia (DRGs) were harvested from neonatal Hannover Wistar rat pups (passage 3) and collected in HBSS with 1% Pen/Strep and 1% amphotericin B. For the dissociation, the isolated DRGs were incubated in a dissociation solution for 40 min within a water bath at 37 °C. After 20 min, collagenase IV (Worthington Biochemical Corporation, Troisdorf, Germany) was added. The DRGs were separated mechanically with a fire polished glass Pasteur pipette (BRAND GmbH + Co. KG, Wertheim, Germany) every 10 min. The digestion was stopped by adding the same amount of attachment medium consisting of N2-Medium (GIBCO, Thermo Fisher Scientific, Waltham, MA, USA) supplemented with 3% FCS. The cell solution was then transferred into a petri dish to allow a controlled seeding of single DRGs on coverslips or scaffolds under a stereomicroscope Stemi SV 6 (Carl Zeiss AG, Jena, Germany).

#### 5.8.2. Analysis of Neurite Outgrowth

To evaluate the influence of the scaffolds on sensory neurite outgrowth behaviour, 2–3 DRGs in sister cultures were seeded on P-ORN-laminin coated coverslips in 300 µL attachment medium (control) and on the scaffolds in a 24-well plate. The scaffolds were washed, fixed, and dried as described in Section 5.7.2. To prevent dehydration of the DRGs on the scaffolds during the pre-incubation time of 60 min at 37 °C and 5% CO_2_, a 50 µL drop of the medium was placed on top of each DRG every 15 min. Afterwards the wells were filled up to a total volume of 1 mL. After 24 h, the medium was changed to a specific culture medium (N2-Medium with 50 ng/mL Nerve growth factor (NGF, Invitrogen, Darmstadt, Germany)) to induce neurite outgrowth. The DRGs were fixed on day 6 with 4% paraformaldehyde (PFA, Sigma Aldrich, Munich, Germany) for 30 min.

### 5.9. Immunofluorescence Analysis

The Schwann cells were cultivated for a total period of 6 days, fixed and stained at specific time points to observe differences in their morphology. On days 3 and 6, the cells on the culture plates, coverslips, and scaffolds were fixed with 4% PFA for 15 min and washed three times with PBS. After blocking unspecific bonds with a 0.3% Triton-X-100 (Sigma-Aldrich Chemie GmbH, Taufkirchen, Germany) and 3% normal goat serum blocking solution (NGS, GIBCO, Thermo Fisher Scientific, Waltham, MA, USA) in PBS for 20 min at room temperature (RT), the nrSCs and ahSCs were incubated overnight at 4 °C with a Schwann cell specific rabbit α-S100 antibody (Dako Denmark A/S, Glostrup, Denmark). The Alexa 488 labelled goat rabbit secondary antibody (Invitrogen, Thermo Fisher Scientific, Waltham, MA, USA) (1:400 in PBS) was added for 1 h after washing three times with PBS at RT. After counterstaining all cell nuclei with 4′,6-Diamidine-2′-phenylindole dihydrochloride (DAPI, 1:1000 in PBS) for 10 min at RT the coverslips and scaffolds were mounted on the slides with Mowiol mounting medium (Sigma-Aldrich Chemie GmbH, Taufkirchen, Germany). The images were acquired with the Cell P software Version 3.4 (Olympus Europa SE & Co. KG, Hamburg, Germany) using an IX70 fluorescence microscope (Olympus Europa SE & Co. KG, Hamburg, Germany).

For the evaluation of neurite outgrowth, the fixed DRGs on the coverslips or scaffolds were incubated with a PBS/0.3% Triton-X-100/3% NGS blocking solution for 1 h at RT and incubated overnight (4 °C) with neuron specific mouse α-β-III-tubulin antibody (Abcam plc, Cambridge, UK) (1:500) in PBS/0.3% Triton-X-100/1% NGS. DRGs were washed three times with PBS and incubated afterwards with Alexa 555-labelled goat mouse secondary antibody (Invitrogen, Thermo Fisher Scientific, Waltham, MA, USA) (1:400 in PBS) for 1.5 h at RT. The coverslips and scaffolds were mounted on microscopic slides as described before. The experiment was performed three times. Whole DRGs with neurites were captured using a BX51 fluorescence microscope (Olympus Europa SE & Co. KG, Hamburg, Germany) and the Stereo Investigator software (MBF Bioscience, Williston, VT, USA) with multiple image alignment and Z-stacks. Since not all DRGs attached firmly to the scaffolds and not all the attached ones demonstrated robust neurite outgrowth only a qualitative monitoring could be performed.

### 5.10. Statistical Analysis

All statistical analyses were performed in OriginPro 2021 (OriginLab Corporation, Northampton, MA, USA). A Shapiro–Wilk test was used to analyse the normality of the data. If normal, statistical significances were analysed using one-way ANOVA (*p* = 0.05) followed by a Tukey’s multiple comparison test. For not normally distributed data, Kruskal–Wallis ANOVA with Dunn’s post hoc test was performed to analyse statistical significances. The values at *p* < 0.05 were considered significantly different.

## Figures and Tables

**Table 2:** The results of the peak deconvolution in the wavenumber range of 740–930 cm^−1^.

	Main Characteristics of the Bands (Mean ± SD, n = 6)									
	797 cm^−1^ (α PVDF)			839 cm^−1^ (β PVDF)			845 cm^−1^ (β P(VDF-TrFE))			β/α Ratio
	Intensity, arb. un.	FWHM, cm^−1^	Area, %	Intensity, arb. un.	FWHM, cm^−1^	Area, %	Intensity, arb. un.	FWHM, cm^−1^	Area, %	Based on Equation (3)
PVDF raw	0.8 ± 0.1	11.7 ± 0.1	29.6 ± 2.6	0.3 ± 0.1	12.8 ± 0.2	10.9 ± 3.1	-	-	-	0.4 ± 0.1
PVDF mat	0.4 ± 0.1	13.0 ± 0.4	11.8 ± 1.4	0.8 ± 0.1	13.4 ± 0.5	27.6 ± 6.1	-	-	-	2.3 ± 0.6
P(VDF-TrFE) raw	-	-	-	-	-	-	0.9 ± 0.1	20.1 ± 0.1	52.6 ± 0.6	-
P(VDF-TrFE) mat	-	-	-	-	-	-	0.9 ± 0.1	21.0 ± 0.4	47.1 ± 1.6	-

**Table 3:** Optimal parameters for the manufacturing of the scaffolds.

Scaffold Type	Concentration, wt%	Needle, Gauge	Applied Voltage, kV	Spinning Distance, cm	Flow Rate, mL/h	Collector Rotation Speed, rpm
PCL	17	21G	15	17	4	500
PVDF	10	27G	12	16	2.5	500
	15		15	16		
	20		12	16		
P(VDF-TrFE)	10	21G	24	26	2.5	500
	15		20	17		
	20		15	17		

## Data Availability

All reported data that support the findings of this study are in the manuscript and Supplementary Materials. The raw data are available from the corresponding author upon reasonable request.

## Supplementary Materials

The following are available online at https://www.mdpi.com/article/10.3390/ijms222111373/s1.
