# Supplementary material for: PVDF and P(VDF-TrFE) Electrospun Scaffolds for Nerve Graft Engineering: A Comparative Study on Piezoelectric and Structural Properties, and In Vitro Biocompatibility
Source: Int J Mol Sci. 2021 Oct 21;22(21):11373. doi: 10.3390/ijms222111373 (PMC8583857; doi:10.3390/ijms222111373)
Supplement: Supplementary file 1 [file ijms-22-11373-s001.zip › ijms-1410933-supplementary.pdf]

International Journal of *Molecular Sciences*

MDPI

Article

PVDF and P(VDF-TrFE) electrospun scaffolds for nerve graft engineering: a comparative study on piezoelectric and structural properties, and in vitro biocompatibility

Oleksandr Gryshkov [1,2,*,+], Fedaa AL Halabi [1,*,+], Antonia Isabel Kuhn [1], Sara Leal-Marin [1,2], Lena Julie Freund [3], Maria Förthmann [3], Nils Meier [4], Sven-Alexander Barker [1,2], Kirsten Haastert-Talini [3,$], Birgit Glasmacher [1,2,$]

Supplementary information

1. Representative results of the deconvolution of the Raman spectra

Representative results of the deconvolution of the Raman spectra in the region of 740-930 cm^{-1} for the PVDF pellet (Figure S1), PVDF fibre mat (Figure S2), P(VDF-TrFE) powder (Figure S3) and P(VDF-TrFE) fibre mat (Figure S4).

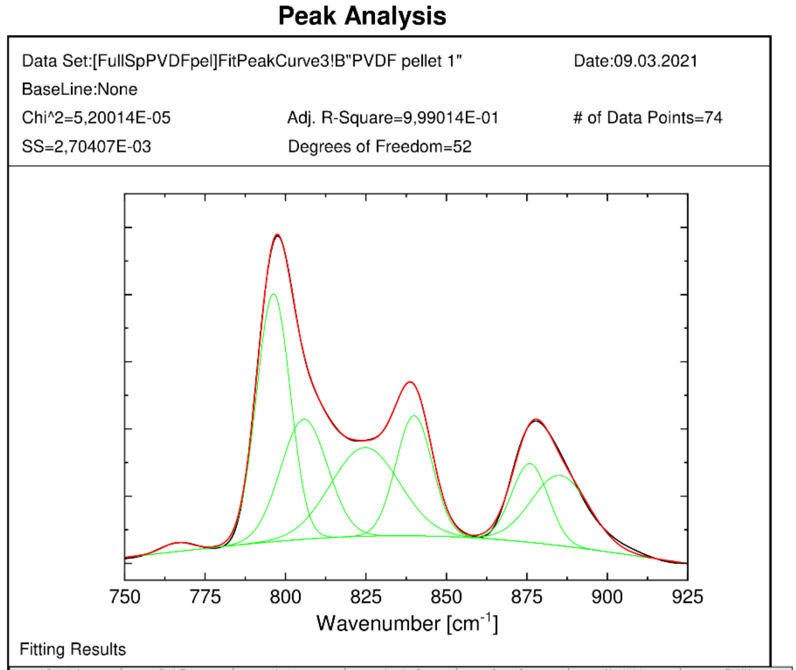

Peak Index	Peak Type	Area Intg	Area IntgP	Center Grvty	Max Height	FWHM
1	Gaussian	0,30913	0,84687	766,31437	0,02857	10,16606
2	Gaussian	9,6212	26,35753	796,23378	0,7726	11,69883
3	Gaussian	6,50291	17,81489	805,72598	0,36545	16,71639
4	Gaussian	7,01367	19,21412	824,68518	0,26689	24,68724
5	Gaussian	5,03296	13,78793	839,96456	0,37117	12,73847
6	Gaussian	3,33168	9,12721	876,01395	0,24326	12,86642
7	Gaussian	4,69113	12,85146	885,4013	0,21223	20,76505

Figure S1. Results of the Gaussian deconvolution of the Raman spectra for the 20wt% PVDF pellet in the region of 740-930 cm^{-1}.

Peak Analysis

Data Set:[FullSpPVDFmat]Sheet1!F"PVDF mat 5" Date:09.03.2021
BaseLine:None
Chi^2=3,37202E-05 Adj. R-Square=9,99444E-01 # of Data Points=74
SS=1,85461E-03 Degrees of Freedom=55

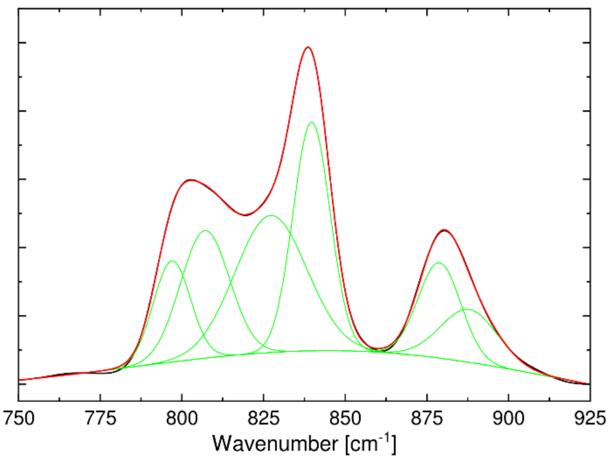

Fitting Results

Peak Index	Peak Type	Area Intg	Area IntgP	Center Grvty	Max Height	FWHM
1	Gaussian	4,21586	10,61152	796,92459	0,30788	12,86398
2	Gaussian	7,03883	17,71705	807,04073	0,38193	17,31356
3	Gaussian	10,98768	27,65647	827,19209	0,40551	25,45484
4	Gaussian	9.53354	23,99634	839.6944	0,69566	12,87438
5	Gaussian	4,56475	11,48968	878,85554	0,28767	14,90697
6	Gaussian	3,38848	8,52895	887,96003	0,15716	20,25536

Figure S2. Results of the Gaussian deconvolution of the Raman spectra for the 20wt% PVDF fibre mat in the region of 740-930 cm^{-1}.

Peak Analysis

Data Set:[FullSpTrFEp]Sheet1!G"TrFE powder 6" Date:09.03.2021
BaseLine:None
Chi^2=1,43664E-05 Adj. R-Square=9,99743E-01 # of Data Points=74
SS=8,33254E-04 Degrees of Freedom=58

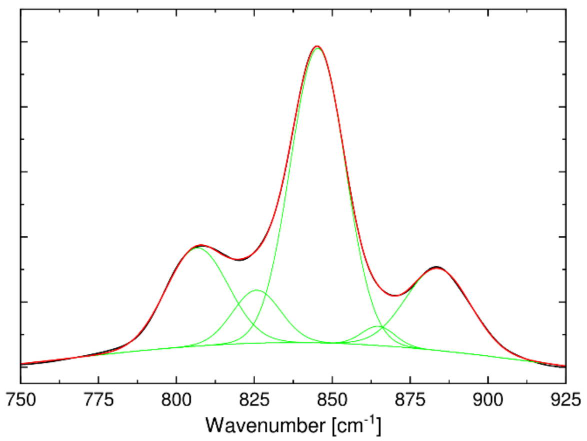

Fitting Results

Peak Index	Peak Type	Area Intg	Area IntgP	Center Grvty	Max Height	FWHM
1	Gaussian	7,52169	19,80961	806,61055	0,30518	23,15383
2	Gaussian	3,12334	8,22583	825,71148	0,16602	17,67386
3	Gaussian	20,01874	52,72263	845,40255	0,9192	20,45938
4	Gaussian	0,78337	2,06315	864,84374	0,06181	11,90549
5	Gaussian	6,52277	17,17878	884,00601	0,25531	24,00077

Figure S3. Results of the Gaussian deconvolution of the Raman spectra for the 20wt% P(VDF-TrFE) powder in the region of 740-930 cm^{-1}.

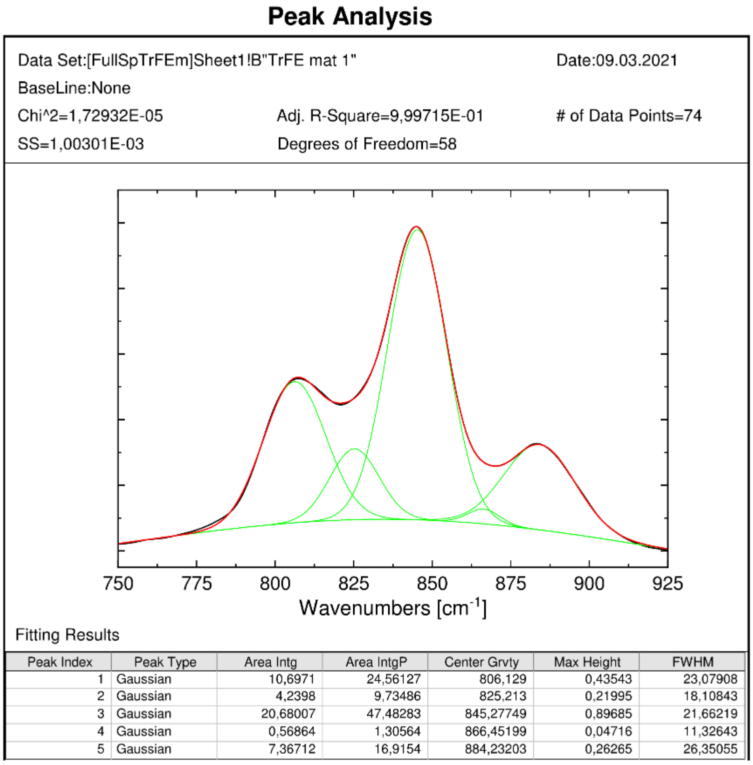

Peak Index	Peak Type	Area Intg	Area IntgP	Center Grvty	Max Height	FWHM
1	Gaussian	10,6971	24,56127	806,129	0,43543	23,07908
2	Gaussian	4,2398	9,73486	825,213	0,21995	18,10843
3	Gaussian	20,68007	47,48283	845,27749	0,89685	21,66219
4	Gaussian	0,56864	1,30564	866,45199	0,04716	11,32643
5	Gaussian	7,36712	16,9154	884,23203	0,26265	26,35055

Figure S4. Results of the Gaussian deconvolution of the Raman spectra for the 20wt% P(VDF-TrFE) fibre mat in the region of 740-930 cm^{-1}.

2. Development and validation of the dynamic impact machine

2.1. Development of the measurement device

The presented measurement method is analogous to the Berlincourt method and is illustrated in Figure 10A,C (main manuscript). The developed method consists of a measurement chamber, the solenoid drive BOSE ElectroForce LM1 TestBench, a measurement amplifier, as well as the acquisition, processing and digitalisation of the obtained data with a programmed software. The set-up and function of each component will be described in the following.

2.1.1. Measurement chamber

The measurement chamber is surrounded by a metal housing. Therefore, the measurement within the chamber is shielded from environmental interfering electric and magnetic fields and allows an undisturbed signal acquisition.

The set-up of the measurement chamber is schematically shown in Figure 10B (main manuscript). Firstly, the load cell (1) has to be fixed onto the bracket (2) which is the static counterpart of the BOSE solenoid drive (3). Following, the measurement chamber (4) is bolted onto the bracket. Thereby, the load cell is situated within a manufactured recess inside the chamber. After opening the chamber, and dependent upon whether d_{33} or d_{31} respective d_{32} shall be measured, the 1st electrode (5) is screwed onto the load cell. A 2nd electrode (6) connects to the solenoid drive via a threaded pin. Within the chamber the drive is moved to its maximum extremity. The 2nd electrode is positioned within the corresponding recess. At a suitable measurement distance, the drive and the bracket are bolted onto a metal table. Finally, the electrodes are connected to the BNC-plug, which is located in the lid of the measurement chamber, via banana plugs.

2.1.2. Electrodes

Figure S5 shows the different electrodes which were used for the measurement of the piezo-module, which is also known as the piezoelectric charge constant. Two different

pairs of electrodes can be utilized for the determination of d_{33}–a punctual and a flat load geometry, respectively. A punctual load is preferred for relatively thick as well as mechanically stable samples. In this case the sample is not laterally clamped and can therefore elongate without any additional resistance. A surface load is preferred for thin and susceptible samples (such as electrospun scaffolds). This leads to a reduced mechanical tension onto the loaded samples. At the same time the lateral elongation is inhibited, inducing an additional load value and increasing the measured signal [84]. For the measurement of d_{31} and d_{32}, the sample has to be laterally elongated. Therefore, clamping electrodes were produced and fixed onto the edge of the samples. In order to enable the discharge during the induced tensile load via the lateral clamping electrodes, the sample surface has to be conductive.

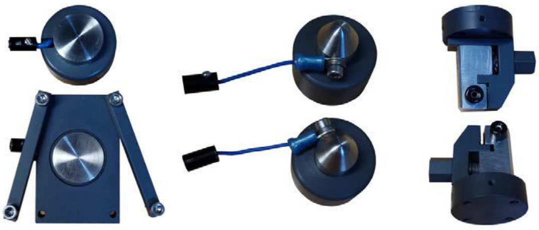

Figure S5. Electrodes: measurement of d_{33} with a surface load (left) and punctual load (centre); measurement of d_{31} (right).

2.1.3. Measurement amplifier

Not the induced voltage and sample capacitance, but the induced charge is measured for the determination of the piezoelectric module through the dynamic measurement method. Besides the reduced measuring expenditure, the parasitic capacitance can be excluded. This can be attributed to the amplification circuit which will be further described in the following. Figure S6 shows the amplification circuit within its casing.

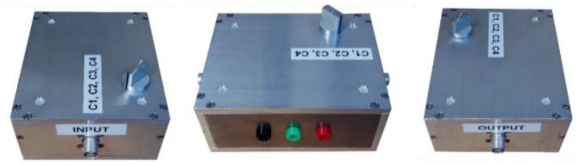

Figure S6. Views of the final assembly of the amplification circuit within the casing.

Principle of the signal processing

PVDF is a dielectric and has, dependent upon the geometry, a high output resistance in the rage of several TΩ. Therefore, a direct measurement of the induced voltage with an oscilloscope, which has an input resistance of several MΩ, would be incorrect. The generated charge would not stay attached to the electrodes and would therefore flow towards the lower resistor of the oscilloscope, immediately. The aim of the developed signal processing system is to simulate a high input resistance for the piezoelectric material. Additionally, the availability of a low output resistance for the measurement system shall be included.

An LF355 operational amplifier from Texas Instruments was utilized for the signal processing in a JFET19 (Junction FET) configuration. This amplifier has a high differential input resistance between the inverted and non-inverted inputs, a low input current, and a high voltage amplification. In general, all operational amplifiers with these properties are applicable (JFET or COMS operational amplifiers). The signal processing is based upon a capacitive feedback differential amplifier, in which the parasitic capacitance C_l of the cables has no influence on the signal [83]. Figure S7 demonstrates the circuit diagram

of the amplification. Here, the induced current I_q of the piezoelectric sample is fully compensated by the feedback current I_f. Thereby, the capacitor C_f is equally charged to the induced charge of the piezoelectric sample. Regarding the feedback circuit, a resistor is connected in parallel to the capacitor C_f by which a continuous discharge of C_f is ensured.

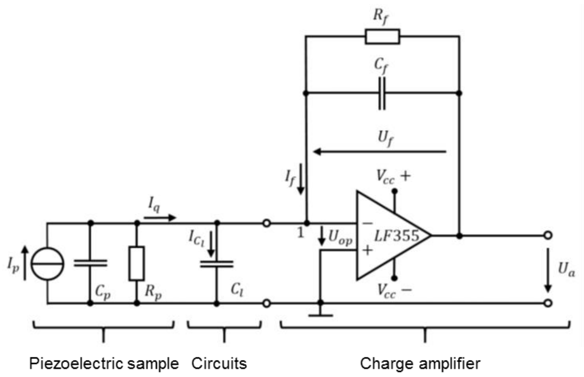

Figure S7. Circuit diagram of the measurement amplifier.

In order to reconstruct the charge of the feedback capacitor C_f and under consideration of the definitions $I = dQ/dt$ and $Q = U \cdot C$, the following Equation S1 describes the current at the electrical node 1 shown in Figure S7:

$$I_q - I_{C_l} - I_f = I_q - C_l \cdot \frac{dU_{op}}{dt} - C_f \cdot \frac{dU_f}{dt} = 0\,. \tag{S1}$$

The voltage U_{op} between the amplifiers' inputs is equal zero as a consequence of the high input resistance. Therefore, the electrical node 1 is situated at the virtual mass. Thus, there is no voltage development at the parasitic capacitance C_l, and therefore no current flow is detectable. In this case the Equation S1 simplifies to the following Equation S2:

$$I_q = C_f \cdot \frac{dU_f}{dt}, \tag{S2}$$

or via a time-dependant integration, and under consideration of $U_f = -Ua$ (Equation S3):

$$Q = -U_a \cdot C_f\,. \tag{S3}$$

Principally, the measurement amplifier can be viewed as a charge-to-voltage converter which converts the charge from the piezoelectric material into a proportional voltage U_a at the measurement capacitor C_f. The derivation indicates that the output voltage U_a of the amplifier is only dependent upon the supplied charge Q as well as the feedback capacitance C_f. Parasitic capacitances C_l have no influence.

Dimensioning and simulation of the measurement amplifier

The components of the electric circuit, C_f and R_f, have to be suitably dimensioned in order to allow the setting of the amplification within the correct effective range. The dimensioning was carried out on the basis of the PVDF fibre mats which were produced out of a 20% polymeric solution. For the further dimensioning the fibre mats are viewed as a solid material. (The sample geometry was assumed to be an ideal solid material with a constant thickness. The actual samples contain pores and possess an inhomogeneous thickness. In theory, a reduction of the effective sample capacitance and the resistance should be obtained.) The modelling of the piezoelectric film can be viewed in Figure S8. Herein, the film can either be modelled as a current supply with a parallel capacitor C_p and a resistor R_p or as a voltage supply with a serial connection of the elements.

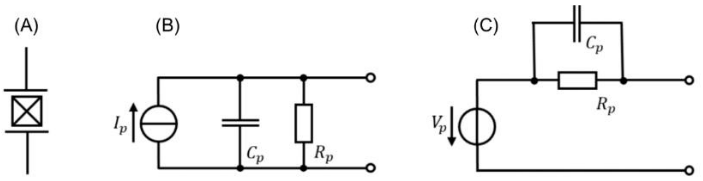

Figure S8. Modelling of the piezoelectric materials [82]. (**A**) Schematic symbol; (**B**) with current supply; (**C**) with voltage supply.

The sample can be viewed as a plate capacitor. Therefore, the electric resistance and the capacitance can be calculated via the Equations S4 and S5, respectively. Thus, the utilized sample for the dimensioning of the components has a resistance R_p of 4.13×10^{13} Ω and a capacitance C_p of
2.57×10^{-10} F:

$$R_p = \rho_e \cdot \frac{t}{A}, \tag{S4}$$

$$C_p = \varepsilon_0 \cdot \varepsilon_r \cdot \frac{A}{t}. \tag{S5}$$

As shown in Figure S9, a linear correlation of the induced voltage during the mechanical loading is obtainable, assuming a piezoelectric module d_{33} of the lead-zirconate-titanate (LZT) ceramics of 33×10^{-12} C/N.

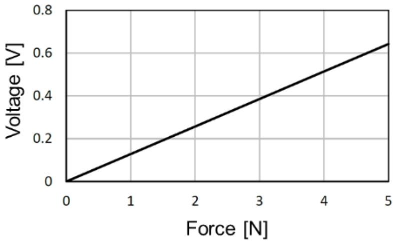

Figure S9. Linear correlation between the applied force and induced voltage of the 20w% PVDF fibre mat under specific assumptions.

As illustrated in the circuit diagram of the measurement amplifier, the capacitance of the capacitor C_f acts as the measurement sensitivity under a constant charge Q. The measured signal increases with a decreasing dimensioning of the capacitance C_f (see Equation S3). The value of the resistance R_f defines the discharge time of the capacitor C_f. In theory, in the event of $R_f \rightarrow \infty$ the measurement capacitor C_f cannot discharge. Therefore, the output voltage U_a would be available at any time. In order to receive a relatively high and prolonged measured signal, a relatively low capacitance C_f of 1×10^{-10} F and a relatively high resistance of 2×10^{9} Ω were selected. Figure S10 illustrates the simulation of the amplifying circuit with the chosen values.

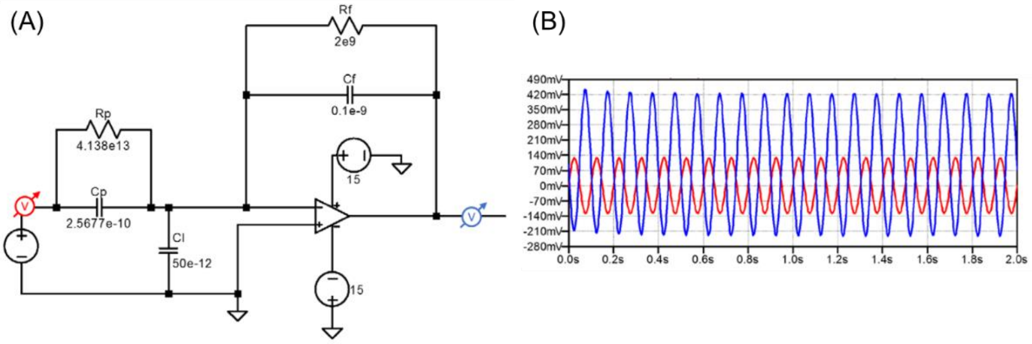

Figure S10. Simulation of the amplifying circuit. (**A**) Circuit. (**B**) Red signal: induced voltage at the piezoelectric material; blue signal: voltage at the measurement capacitor.

The simulation was performed with LTSpice. A loading of the PVDF fibre mat with 1 N corresponds to an induced voltage of 0.129 V (Figure S9), under consideration of the defined assumptions. The simulation of a 10 Hz sinusoidal loading of the sample with 1 N was therefore represented by a sinusoidal voltage with a maximum of |0.129 V| (red signal in Figure S10). The blue signal represents the voltage at the measurement capacitor C_f. Within a steady state and under consideration of an offset of 97 mV, which originates due to the high resistance R_f and the effective input current of the operational amplifier, a positive voltage of 0.129 V is measured at the sample and a negative voltage of 0.33 V is detected at the capacitor C_f. A feedback charge of 33×10^{-12} C is detected in the case of a sample loading. Further, a feedback charge of -33×10^{-12} C is obtained for the unloading of the sample, when multiplying the voltage with a capacitance value of the measurement capacitor C_f of 1×10^{-10} F, and in correlation to Equation S3. A piezoelectric module of $\pm33\times10^{-12}$ C/N is obtained under the consideration of a mechanical load of 1 N. A switchable capacitor C_f was selected in order to utilize the measurement amplifier in a wider effective range: C_{f1} (1×10^{-10} F), C_{f2} (1×10^{-9} F), C_{f3} (1×10^{-8} F), C_{f4} (1×10^{-7} F).

Time response of the measurement signal

An ideal operational amplifier in conjunction with a parallel capacitor and a resistor can be viewed as an 1st order active high-pass [81]. The differential is (Equation S6):

$$\frac{dU_a}{dt}+\frac{U_a}{R_f\cdot C_f}=-\frac{dQ}{dt}\cdot\frac{1}{C_f}. \tag{S6}$$

The respective transfer function is (Equation S7):

$$U_a=\frac{-j\cdot\omega\cdot q\cdot R_f}{1+j\cdot\omega\cdot R_f\cdot C_f}. \tag{S7}$$

Figure S11 illustrates the amplitude response of the transfer function for the included capacitors $C_{f1\text{-}4}$. In correlation with an increasing frequency, the attenuation of the signal is 20 dB/decade until the cut-off frequency f_c is reached.

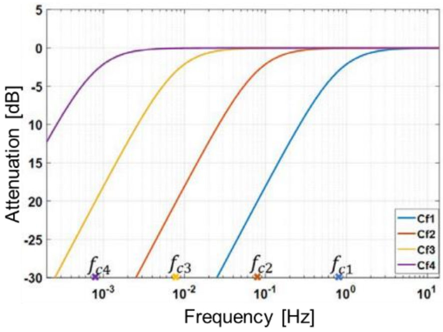

Figure S11. Attenuation of the measurement system with different capacitance values.

Thus, measurements are only feasible above the cut-off frequency. As shown in Equation S8, a calculation can be accomplished via the definition of a 1st order high-pass filter:

$$f_c=-\frac{1}{2\cdot\pi\cdot R_f\cdot C_f}. \tag{S8}$$

Possible measurement frequencies for the included capacitors $C_{f1\text{-}4}$ are as follows: C_{f1} from 0.8 Hz, C_{f2} from 0.08 Hz, C_{f3} from 0.008 Hz and C_{f4} from 0.0008 Hz. Measurements of a near static state can be conducted with an increasing capacitance C_f due to the relatively

high resistance R_f of 2×10[9] Ω. It has to be noted that the measurement sensitivity decreases when the capacitance is increased.

Validation of the measurement amplifier

A piezoelectric transducer was utilized for the validation of the charge measurement which was performed with the described measurement amplification circuit. The piezoelectric transducer consists of a both-sided metallised LZT piezo-ceramic. Thereby, one side is coated with conductive particles and the other side is fixed on a metal plate (Figure S12).

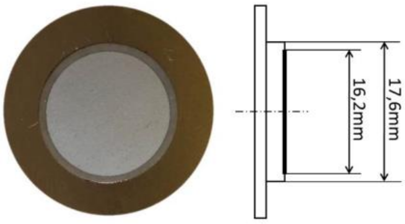

Figure S12. Piezo LZT ceramics utilized for the validation of the measurement amplifier.

The piezoelectric module d_{33} can be calculated via the following Equation S9:

$$d_{33} = \frac{Q}{F} = \frac{w \cdot U \cdot C_p}{W \cdot F}, \quad \text{(S9)}$$

with Q being the induced charge, C_p is the capacitance of the sample, w is the area of the mechanical load, W is the area of the electrodes, and F is the loading force.

Under consideration of the piezoelectric module d_{33} shown in Equation 1, the induced charge during the mechanical load is defined via the following Equation S10:

$$Q = U \cdot C_p \cdot \frac{w}{W}, \quad \text{(S10)}$$

with w/W being the areal factor of the LZT ceramics equal to 1.08.

The charge measurement was partly conducted with the designed measurement amplifier, and partly with the mathematical product of the voltage, capacitance and areal factor. The results were compared. In order to obtain an accurate determination of the voltage under utilization of the oscilloscope, it has to be ensured that the measured signal is located at the electrodes and does not equalize via the oscilloscopes' resistor. Therefore, a high input resistance of the oscilloscope has to be applied, compared to the output resistor at the measured signal. Within the measurement chamber, the LZT ceramic was placed between the electrodes. During the measurement a resistance R_{LZT} of 500 kΩ and a capacitance C_{LZT} of 22 nF was detected at the connecting cables. The utilized probe of the oscilloscope possesses an input resistance of 10 MΩ. The systemic measurement deviation is 0.05 and can be neglected. Furthermore, the parasitic capacitances of the electrical leads are in the order of pF and have therefore a neglectable influence on the capacitance of the ceramic. Figure S13A illustrates the results of both charge measurements over a frequency range of 1–50 Hz. In view of the differently valued measurement capacitors, the measurement of the obtained charge of the measurement amplifier was carried out repeatedly.

Regarding the LZT ceramic, the lowest measurement capacitor C_{f1} could only be applied for the lower frequency range of up to 5 Hz. For higher frequencies, the amplification via C_{f1} exceeded the supply voltage of the operating amplifier. An initial tension of 0.5 N was applied to the LZT ceramic for all measurements and was additionally loaded with 0.25 N over the frequency range.

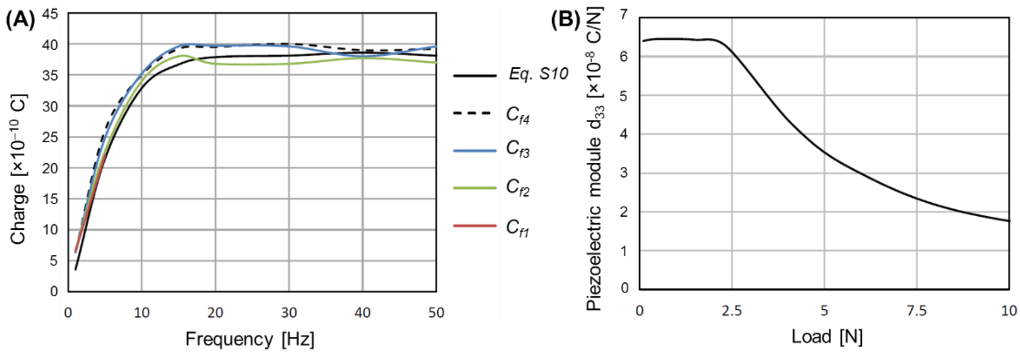

Figure S13. Induced charge (**A**) and influence of the applied force onto the piezo-module (**B**) of the LZT ceramic. (**A**) An initial tension of 0.5 N was applied and additionally loaded with a further 0.25 N. The measurement was performed via the measurement amplifier as well as the product of the induced voltage, sample capacitance and the areal factor. $C_{f1\text{-}4}$ are the respective capacitors. (**B**) The load frequency is 20 Hz.

An approximately equal charge was measured via the product of the induced voltage, sample capacitance and the areal factor, as well as via the measurement amplifier. Both measurement methods showed an equal charge progression when increasing the load frequency. The signal sequence over the frequency can be referred to the time response of the samples' signal which is defined as the following Equation S11:

$$\tau_{LZT} = R_{LZT} \cdot C_{LZT} = 0.011s \,. \tag{S11}$$

Thus, the equivalent load frequency can be calculated as Equation S12:

$$1/(2 \cdot \pi \cdot \tau_{LZT}) = 14.47 Hz \,. \tag{S12}$$

Below the load frequency a rapid discharge occurs via the internal sample resistance. Thus, only a fraction of the charge is detected. The measured signal is obtained in its entirety above this frequency.

Figure S13B represents the influence of the mechanical load onto the piezo-module d_{33} at a constant load frequency of 20 Hz. Up to 2.4 N, the LZT ceramic exhibits a linear correlation between the mechanical and the electrical quantities, and proves therefore the constant value of the piezo-module. The LZT ceramic shows a non-linear character with an increasing load. The progression in charge is not proportional to the loading, by which a reduction of the piezo-module seems to appear with an increase in load.

Finally, a non-piezoelectric cellulose sample served as a negative control. Therefore, the identical measurement parameters were set as compared to the LZT ceramic. No progression in charge was measured at the surface of the cellulose during the variation of the frequency while maintaining a constant force, as well as vice versa.

Voltage supply of the measurement amplifier

The operating voltage V_{CC} of the included operational amplifier is ±15 V, as described in the respective data sheet. For this purpose a power supply unit with the required symmetric supply voltage was developed, analogous to [80]. Figure S14 illustrates the electrical circuit diagram (Figure S14A) as well as the fabricated housing of the power supply unit (Figure S14B). The measurement amplifier is connected to the power supply unit via banana plug cables.

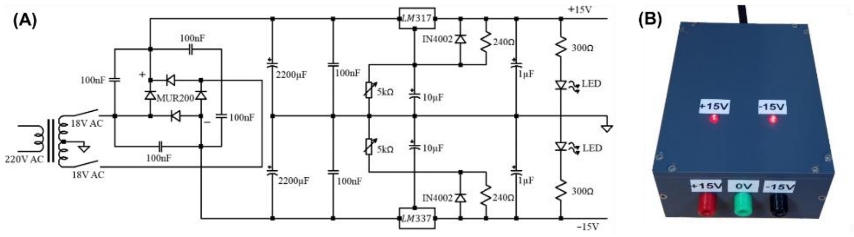

Figure S14. The electrical circuit diagram (**A**) and fabricated symmetric ±15 V DC power supply unit (**B**).

The utilized transformer has two independent output windings of which each transforms the 220 V supply voltage into an 18 V alternating voltage. When both windings are connected in serial, the connection between them can be viewed as the referring ground potential. Both 18 V alternating voltages are connected to the input alternating voltages of the bridge rectifier circuit. A positive direct voltage of 18 V is obtained at the positive output of the bridge rectifier circuit, and a negative 18 V direct voltage at the negative output, respectively. Capacitors with a capacitance of 100 nF are included in parallel to each rectifier. These elements prevent interfering radiation which is generated by the individual rectifiers. Two 2200 µF electrolytic capacitors were included for the smoothing of the direct voltages. In order to regulate and stabilize the symmetric direct voltage, the voltage regulator LM317 was utilized for the positive direct voltage and the voltage regulator LM337 for the negative direct voltage, respectively. If connected to the adjustable potentiometer 3296 with 5 kΩ, a regulation of the symmetric voltage in the range of 1.2-37 V is theoretically feasible. As described within the respective data sheet, the voltage regulators are equipped with a 100 nF capacitor, a 10 µF and a 1 µF electrolytic capacitor, a 240 Ω resistor, and an IN4002 protective rectifier diode. At the other end of the circuit a status LED is included with a pre-inserted protective resistor of 300 Ω.

2.2. Data processing

The software WinTest7 controls the magnitude of the mechanical load, the type of load as well as the load frequency at the BOSE solenoid drive. During the measurement the effective load applied onto the sample is saved as individual data points within a text file.

The measurement amplifier provides a low resistance output signal which enables an accurate measurement with standard measurement technology. Here, the capturing of the voltage at the measurement capacitor of the measurement amplifier was performed with the oscilloscope Siglent SDS1102CML+. The recorded voltage signal is digitalised through the software EasyScopeX and the obtained data points are saved within a text file.

In order to furtherly process the saved data points of the measured voltages at the measurement capacitor as well as of the mechanical load, a program was created via Visual Basic within the Microsoft Excel environment. Figures S15A,B exemplary illustrate the obtained measured points of the oscilloscope and the BOSE system, respectively. The measured points are plotted within the diagram and the turning points are determined under consideration of outliers. Furthermore, the percentage of outliers can be adjusted via arrow buttons. Thus, an averaging of the turning points is implemented (see orange line). Within the BOSE window the load frequency can be determined by placing the green register onto two neighbouring turning points. The appropriate measuring capacitor has to be selected within the calculation window of the piezo-module which corresponds to the set capacitor on the amplifiers' casing.

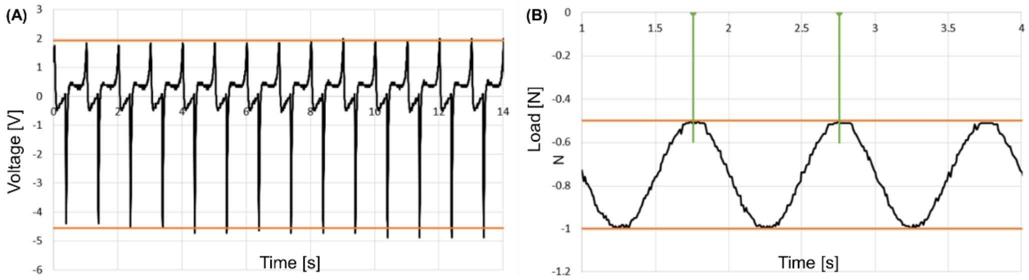

Figure S15. Data acquisition. Measured points with the oscilloscope (**A**) and with the BOSE system (**B**).

2.3. Performing the measurements

As previously mentioned, the sample which is intended for the piezoelectric analysis under a dynamic load, is usually preloaded. For the validation of the measurement amplifier, the LZT ceramic was preloaded between the electrodes. A surface charge of the LZT ceramic could be detected during the oscillating loading. In comparison, no charge could be detected during the oscillating loading in the case of preloaded fibre mats. A charge can be measured if the fibre mat is not preloaded, and the loading occurs as an impulse so that the contact between the electrodes and the sample is fully interrupted between each loading cycle. Therefore, the fibre mat is fixed onto a frame which is pushed onto an electrode (see Figure 11A of the main text). Thus, the fibre mat and the respective electrode are in a permanent contact. A brief contact between the fibre mat and both electrodes can only be observed during the maximum of the load cycle. Subsequently after its assembly, the measurement chamber is connected to the measurement amplifier via BNC-cables. The output of the measurement amplifier can be connected to the oscilloscope via a further BNC-cable. Finally, the power supply unit is connected to the measurement amplifier via three banana plugs.

References

80. Stewart, M.; Battrick, W.; Cain, M. Measuring Piezoelectric d33 Coefficients Using the Direct Method. Available online: https://eprintspublications.npl.co.uk/2768/1/mgpg44.pdf (accessed on 12 April 2021).
81. Dally, J.W.; Riley, W.F.; MacConnell, K.G. *Instrumentation for Engineering Measurements*, 2nd ed.; John Wiley & Sons: New York, NY, USA, 1993; ISBN 9780471551928.
82. Karki, J. Signal Conditioning Piezoelectric Sensors: Mixed Signal Products—Application Report. Available online: https://www.ti.com/lit/an/sloa033a/sloa033a.pdf?ts=1618224294724&ref_url=https%253A%252F%252Fwww.google.com%252F (accessed on 12 April 2021).
83. Aupetit, N. Signal Conditioning for Shock Sensors: Application Note. Available online: https://www.st.com/resource/en/application_note/dm00188713-signal-conditioning-for-shock-sensors-stmicroelectronics.pdf (accessed on 12 April 2021).
84. Narayanan, K. Variable Power Supply Using LM317 Voltage Regulator. Available online: https://electrosome.com/variable-power-supply-lm317-voltage-regulator/ (accessed on 12 April 2021).
